# Pneumo−/enteritis in dromedary camels (*Camelus dromedarius*)

**DOI:** 10.3389/fvets.2026.1908854

**Published:** 2026-09-02

**Authors:** Mohie Haridy, Thamer Othman Alhasyani, Abdulrahman A. Alkheraif, Saleh M. Albarrak

**Affiliations:** Department of Pathology and Laboratory Diagnosis, College of Veterinary Medicine, Qassim University, Buraydah, Saudi Arabia

**Keywords:** disease control, dromedary camel (*Camelus dromedarius*), enteritis, infection, pneumonia, pathology, pathophysiology

## Abstract

Pneumo−/enteritis is a co-occurrence of respiratory and enteric syndrome unique to juvenile weaned camels that causes high morbidities and mortalities and economic losses in food production in Saudi Arabia and the surrounding Gulf countries. This review focuses on its epidemiology, clinical impact, and control strategies in Saudi Arabia and neighboring arid Middle Eastern countries, where camel production is a major livestock sector. Pneumo−/enteritis is a multifactorial disease syndrome characterized by the concurrent or sequential development of pneumonia and enteritis in camel calves. The condition typically results from a combination of infectious agents, including viruses, bacteria, and parasites acting synergistically, often under the influence of predisposing stress factors that compromise immune function. Moreover, this review summarizes current knowledge on the epidemiology, pathogenicity and lesions associated with the major pathogens that have been reported to cause pneumo−/enteritis in dromedary camels. Unlike previous reviews that have examined camel respiratory or enteric diseases as separate entities, this review provides an integrated assessment of pneumo−/enteritis by synthesizing evidence on viral, bacterial, and parasitic co-infections and their combined contribution to disease development in camel calves., with particular emphasis on the circulating pathogens in the Middle East region with priority to those commonly recorded in Saudi Arabia and surrounding arid nations. The current inefficient protocols for diagnosis of infectious pathogens causing pneumo−/enteritis as well as the lack of vaccination protocols need to be highlighted and further future studies on implantation of diagnostic methodologies and vaccination protocols to control this syndrome are mandatory issues. Despite advances in understanding disease causation, major gaps remain in rapid field diagnostics, pathogen-specific surveillance, and effective vaccination strategies, highlighting the need for integrated control programs tailored to camel herds in arid production systems.

## 1 Introduction

### 1.1 Background of pneumo−/enteritis

Pneumo−/enteritis, a condition characterized by the concurrent or sequential occurrence of pneumonia and enteritis, is well-documented across numerous veterinary species. It is particularly significant in young animals, including calves, lambs, and goat kids, where it contributes to substantial morbidity, mortality, and economic loss. In calves, the syndrome is commonly associated with viral pathogens such as bovine coronavirus (BCoV) and adenoviruses, while bacterial agents including *Escherichia coli* frequently act as primary or secondary contributors. One of the earliest descriptions of pneumo−/enteritis dates to 1940 (1), in which the disease was linked to the opportunistic overgrowth of normal respiratory or intestinal microbiota following reductions in host immunity. Predisposing factors reported in early and contemporary studies include low ambient temperature, poor housing conditions, ectoparasitism, and inadequate colostrum intake, all of which impair immune competence and increase susceptibility to combined respiratory and enteric infections.

In goats, pneumo-enteritis affects multiple breeds and manifests clinically with pyrexia, nasal discharge, coughing, greenish diarrhea, bleating, and in severe cases, abortion. Untreated outbreaks may result in mortality rates approaching up to 95%. Common pathological findings include increased erythrocyte sedimentation rate, moderate leukopenia, enteritis with greenish-yellow intestinal contents, gallbladder distension, cardiomegaly, and pneumonia (2). In Egypt, the etiological profile of pneumo-enteritis among lambs and goat kids revealed *Escherichia coli* (70.99%) as the predominant pathogen, followed by *Staphylococcus aureus* (5.34%) and *Salmonella* spp. (3.82%) (3).

### 1.2 Viral infections contributing to pneumo-enteritis in dromedary camels

Viral infections also play a central role in the pathogenesis of pneumo-enteritis and respiratory disease complexes in camels. In Sudan, peste des petits ruminant virus (PPRV) was detected in 45% of sampled pneumonic camel lungs and frequently occurred alongside other respiratory viruses including PIV-3, RSV, BHV-1, BVDV, and adenoviruses (4). In the United Arab Emirates, metagenomic surveys revealed substantial viral diversity in the upper respiratory tract of dromedaries, spanning 13 genera across 10 distinct viral families (5). An outbreak in Ethiopia demonstrated parainfluenza-3 as a primary pathogen, with *Mannheimia haemolytica* acting as a major secondary bacterial pathogen, emphasizing the synergistic interaction between viral and bacterial agents in camel respiratory disease outbreaks (6).

### 1.3 Bacterial infections contributing to pneumo-enteritis in dromedary camels

In dromedary camels, pneumo-enteritis is an increasingly recognized clinical syndrome, particularly among young calves. Affected animals typically exhibit profuse, watery, yellowish diarrhea with a foul odor, inappetence, elevated rectal temperatures, dehydration, respiratory distress, and poor body conditions. *E. coli* and *Proteus* spp. have been implicated in diarrhea, while *Staphylococcus aureus* is frequently associated with respiratory involvement. Parasitic agents including *Balantidium coli* and *Eimeria* spp., may further contribute to disease development (7). Additional studies identified a wide array of bacterial pathogens from camels with pneumo-enteritis, including *Coliforms, staphylococci, Providencia* spp., *Edwardisella* spp., *Proteus* spp., *Kluyvera, Pseudomonas* spp. *and Shigella*, with mixed infections (85.2%) far more common than single agent infections (8) (Radwan et al.). Similar findings were reported in Ethiopia, where *E. coli*, *Salmonella*, and *Enterococcus* spp. showed high prevalence in diarrheic camel calves (9).

Comprehensive reviews of camel respiratory pathogens show consistent isolation of *Staphylococcus aureus*, *Corynebacterium* spp., *Streptococcus pyogenes*, *E. coli*, *Klebsiella pneumoniae, Pseudomonas aeruginosa, Trueperella* (formerly *Arcanobacterium*) *pyogenes, Mannheimia haemolytica, and Pasteurella multocida* (10, 11). Similar bacterial species were recovered from camels with respiratory signs in Assiut, Egypt (12). As for enteric pathogens, neonatal camelids (llamas, alpacas, and camels) commonly exhibit diarrhea associated with coronavirus, rotavirus, *E. coli*, *Cryptosporidium* spp., *Giardia* spp., and *coccidia* (13). More recent molecular studies highlight the highly multifactorial nature of camel calf diarrhea, revealing the presence of multiple bacterial species harboring diverse virulence genes, along with significant alterations in metabolic, oxidative stress, and immune-related biomarkers, which collectively contribute to disease severity and progression (14). Beyond conventional pathogen detection methods, host-response biomarkers may provide valuable tools for assessing disease severity and prognosis in camel infections. Oxidative-stress biomarkers in dromedary camels have highlighted their potential application for predicting clinical outcomes, survival, and mortality. Oxidative biomarkers may offer promising complementary approaches for early diagnosis, monitoring of disease progression, and prognostic evaluation in camel calves affected by pneumo-enteritis (15).

### 1.4 Parasitic infections contributing to pneumo-enteritis in dromedary camels

Parasitic infections represent an important but often underestimated component of camel pneumo-enteritis because they can compromise host immunity, damage mucosal barriers, and increase susceptibility to secondary viral and bacterial infections. Gastrointestinal parasites, including *Eimeria* spp., *Cryptosporidium* spp., gastrointestinal nematodes, and other helminths, may induce enteric inflammation, villous damage, impaired nutrient absorption, and chronic stress, leading to reduced growth and increased vulnerability of young camel calves to concurrent respiratory infections. Recent reviews have emphasized that parasitic diseases significantly affect dromedary camel welfare by causing nutritional depletion, immune dysregulation, and reduced resistance to other infectious agents (16). Epidemiological investigations in camel populations have demonstrated considerable gastrointestinal parasite burdens and associated risk factors, with zoonotic-relevant protozoal infections such as *Cryptosporidium* representing additional concerns for animal and public health (17). In addition, pulmonary and systemic parasitic infections may contribute to respiratory disease severity; for example, lungworm infections such as *Dictyocaulus cameli* can damage respiratory tissues and facilitate secondary bacterial pneumonia. Hemoparasitic infections, including *Trypanosoma evansi*, have also been reported in camel-rearing regions of Pakistan and may contribute indirectly to pneumo-enteritis by inducing anemia, oxidative stress, and immune suppression (18). Furthermore, ectoparasite infestations, particularly tick-associated stress, can trigger systemic inflammatory responses, altered hematobiochemical profiles, and metabolic disturbances in camel calves, potentially reducing resistance to respiratory–enteric infections (19). Therefore, parasitic infections should not be considered isolated conditions but rather important predisposing factors within the polymicrobial framework of camel pneumo-enteritis, where parasite-induced immune compromise facilitates viral persistence, bacterial overgrowth, and increased disease severity under field conditions.

### 1.5 Epidemiological and risk factors for camel pneumo−/enteritis under desert pastoral systems

Although pneumo−/enteritis syndromes are reported in young ruminants such as calves and lambs, dromedary camels experience distinct disease dynamics shaped by their adaptation to arid and semi-arid production environments (20). Desert pastoralism exposes camel herds to extreme temperature fluctuations, prolonged drought conditions, limited feed and water availability, and seasonal nutritional stress, all of which can compromise calf immunity and increase susceptibility to respiratory and enteric infections (21, 22). In addition, traditional camel production systems often involve mixed-species herding with sheep, goats, and other livestock, creating opportunities for cross-species pathogen exchange and complex polymicrobial infections (23, 24). These ecological and management-related factors, combined with seasonal stressors such as weaning, long-distance movement, and climatic transitions, contribute to a unique epidemiological pattern of pneumo-enteritis in camel calves that differs from disease presentations observed in intensively managed cattle and small ruminants (25, 26).

In camel calves, predisposing management, and environmental stressors such as inadequate colostrum intake, cold stress, overcrowding, and nutritional limitations are not only associated with increased disease susceptibility but also contribute to the emergence of complex viral–bacterial co-infections (27–29). Failure of passive immune transfer due to insufficient colostrum consumption reduces circulating maternal antibodies and impairs early-life protection against respiratory and enteric viruses, allowing primary viral infections to facilitate secondary bacterial colonization of the respiratory and gastrointestinal tracts (20, 30–32). Exposure to cold stress and abrupt climatic changes may further suppress mucosal immune defenses, impair mucociliary clearance, and enhance viral replication, thereby increasing the likelihood of concurrent bacterial invasion. Similarly, overcrowding during seasonal aggregation, transport, or intensive herd management increases pathogen transmission pressure through close contact, aerosol exposure, and fecal–oral contamination, promoting the circulation of multiple respiratory and enteric agents within camel herds. Previous studies of young camel diseases have highlighted the frequent detection of mixed infections involving viral, bacterial, and parasitic agents, suggesting that environmental stressors act as important drivers of pathogen co-proliferation rather than independent risk factors alone (30, 33).

Dromedary camels have a distinct pneumo−/ enteritis risk factors epidemiology under desert pastoralism including arid climate, mixed multi-species herding, and seasonal stressors, all are camel-specific risk drivers. Younger camel calves usually have less developed immune systems, making them highly susceptible to viral and bacterial agents causing pneumo−/ enteritis (34, 35). Malnutrition and sudden changes in feed or concentrated diets drastically alter the gastrointestinal environment, allowing opportunistic bacteria to proliferate inducing respiratory and enteric disorders in camel claves (36). Sudden climatic changes such as temperature and increased relative humidity especially in autumn and winter due to rainy weather and poor ventilation in barns, overcrowding as well as constant exposure to dusty/polluted environments weaken respiratory and digestive mucous membranes and increased liability to respiratory manifestation, pneumonia and diarrhea in dromedary camels (37). Rotavirus was detected in 0 to 3-month-old diarrheic camels in Sudan. Higher prevalence of rotavirus infection was noticed during wet season than dry and winter seasons (38).

### 1.6 Rational and objectives

Pneumo-enteritis in camels is a multifactorial syndrome involving viral, bacterial, and parasitic agents, often occurring as mixed infections, and leading to increased mortality and economic losses. Despite the availability of studies describing pneumonia and enteritis separately in camels, limited information is available regarding their concurrent occurrence as pneumo-enteritis and the associated etiological agents. This study is a narrative review based on previously published literature. Relevant articles were retrieved from databases including PubMed, Google Scholar, and ResearchGate using keywords such as “pneumo-enteritis,” “camels,” “pneumonia,” and “enteritis.” Studies focusing on pneumonia and enteritis in camels were selected, with emphasis on those describing viral, bacterial, and parasitic etiological agents. The collected literature was reviewed and organized into three main sections covering viral, bacterial, and parasitic pathogens implicated in the development of pneumo-enteritis in camels.

Taken together, pneumo-enteritis in camels represents a complex and often life-threatening multifactorial disease syndrome resulting from the interaction of viral, bacterial, and parasitic pathogens. This review integrates current knowledge on these etiological agents, with dedicated sections addressing the individual and combined roles of viral, bacterial, and parasitic contributors to pneumonia and enteritis in camels, and highlights their roles in disease pathogenesis, epidemiology, and overall clinical impact. The objectives of this literature synthesis are threefold: (1) to collate and critically evaluate available data on the prevalence and distribution of viral, bacterial, and parasitic pathogens associated with camel pneumo-enteritis across the Middle East, with emphasis on Saudi Arabia and neighboring arid regions; (2) to summarize the pathological features and lesion patterns resulting from combined respiratory and intestinal infections in camel calves, including the interactions between multiple infectious agents; and (3) to identify current gaps in diagnosis, surveillance, prevention, and field-level control strategies affecting pastoral camel production systems. By integrating epidemiological, pathological, and management perspectives, this review aims to provide a comprehensive framework for understanding and controlling pneumo-enteritis in young dromedary camels.

## 2 Overview of viral pathogens contributing pneumo−/enteritis in dromedary camels

### 2.1 Peste des petits ruminants

The PPR virus is a member of the genus Morbillivirus and family Paramyxoviridae. It has been identified in Sub-Saharan Africa, the Arabian Peninsula, Middle Eastern nations, and India. PPR is a veterinary disease of economic importance that causes significant financial losses. Small domestic and wild ruminants have been affected by PPR. Although PPR primarily infects sheep and goats, it can also infect cattle, pigs, African buffaloes, and camels (39). In domestic sheep and goats, PPR causes a very severe illness marked by fever, oral sores, diarrhea, and pneumonia that often ends in the animal’s death. Anorexia, emaciation, with a ruffled hair coat, and diarrhea were observed in affected camels. Sneezing, nasal discharge, and fever (40 °C) have also been documented. Affected camels are usually weak, unable to keep pace with the rest of the herd, and often become recumbent (40).

Clinical findings in PPRV infection include severe fever (40–41 °C), mucopurulent discharges from nares and eyes associated with cough and dyspnea, erosions and ulcerations of the oral cavity, gastroenteritis leading to diarrhoea or constipation, and severe conjunctivitis (39). In camels, clinical PPRV infection and seroconversion continue to be existed from endemic areas in the Middle East, Africa, and Asia. All clinical symptoms, pathological and histological pictures related to the respiratory and digestive systems, have so far been driven to be analogous to those reported in small ruminants (41). Moreover, abattoir studies report PPRV antigen detection in the lungs of slaughtered camels with pneumonic lesions, indicating a history of pneumonia (42).

Camels in Mandera and Wajir Counties of Kenya suffering from PPRV natural infection manifested clinical signs such as inappetence, debilitation, general weakness, and eventually diarrhea, conjunctival injection and conjunctivitis, and ocular-nasal mucopurulent discharges preceding death (40). In contrast, Fakri et al. (43) reported that experimentally infected camels exhibited no clinical signs, and only mild conjunctival congestion was observed. Camels did not have any changes in their behavior or appetite and remained in good health during the 6-week post-infection observation period. This suggests that experimental infection may not replicate the conditions of natural infection, which is often influenced by environmental or physiological stressors.

Co-housing camels with sheep and goats may facilitate virus transmission and the spread of PPRV among susceptible species (44). In four flocks of sheep and goats herded alongside camel, 13.75% out of 80 individuals presented clinical signs of PPRV infection such as pyrexia, oculo-nasal discharges with or without diarrhea (40). Additionally, Hemida and Al-Ghadeer (45) stated that direct connection of infected and naïve animals promotes transmission of PPRV through both fecal-oral and respiratory pathways. However, Al-Dubaib (46) in a survey of ruminant morbillivirus in sheep, goats, camels, and cattle in Saudi Arabia reported that camels and cattle grazing with infected small ruminants tested negative for PPRV, suggesting that small ruminants do not always play a role in cross-species transmission.

In a serological survey of camel lungs in a slaughterhouse setting for PPRV detection, animals were examined clinically, grossly and serologically (47). Most of camels had no pulmonary aliments at ante-mortem, while few exhibited mild to moderately severe respiratory signs, including nasal discharge, higher respiratory rate, and severe cachexia. Post-mortem examination, regardless of clinical status, revealed pulmonary lesions including hyperemia, fragility, dark discoloration, and tissue thickening, and, in some cases, multiple abscesses (47). Among the 100 pneumonic camel lung samples tested for the presence of PPRV antigen using the hemagglutination assay, 98 samples (98%) tested positive, whereas only 2 samples (2%) were negative (47).

Retrogressive and necrotic lesions in the lymphoid tissues and epithelial cells of the respiratory and gastrointestinal tracts are hallmarks of PPRV pathogenesis. Pyrexia, oculo-nasal discharges, necrotizing and erosive stomatitis, gastroenteritis, diarrhea, and bronchopneumonia make up the main symptoms (48). Histopathology of camel lungs commonly reveals alveolar dilatation, emphysema, thickened alveolar septa, and mononuclear cell infiltration (39). A higher prevalence of respiratory distress was also reported in camels in Jijiga and Afar in Ethiopia. A seroprevalence of PPRV antibodies using ELISA test reached 10% in Afar camels, whereas no antibodies were confirmed in Jijiga (49).

**Figure 1:**
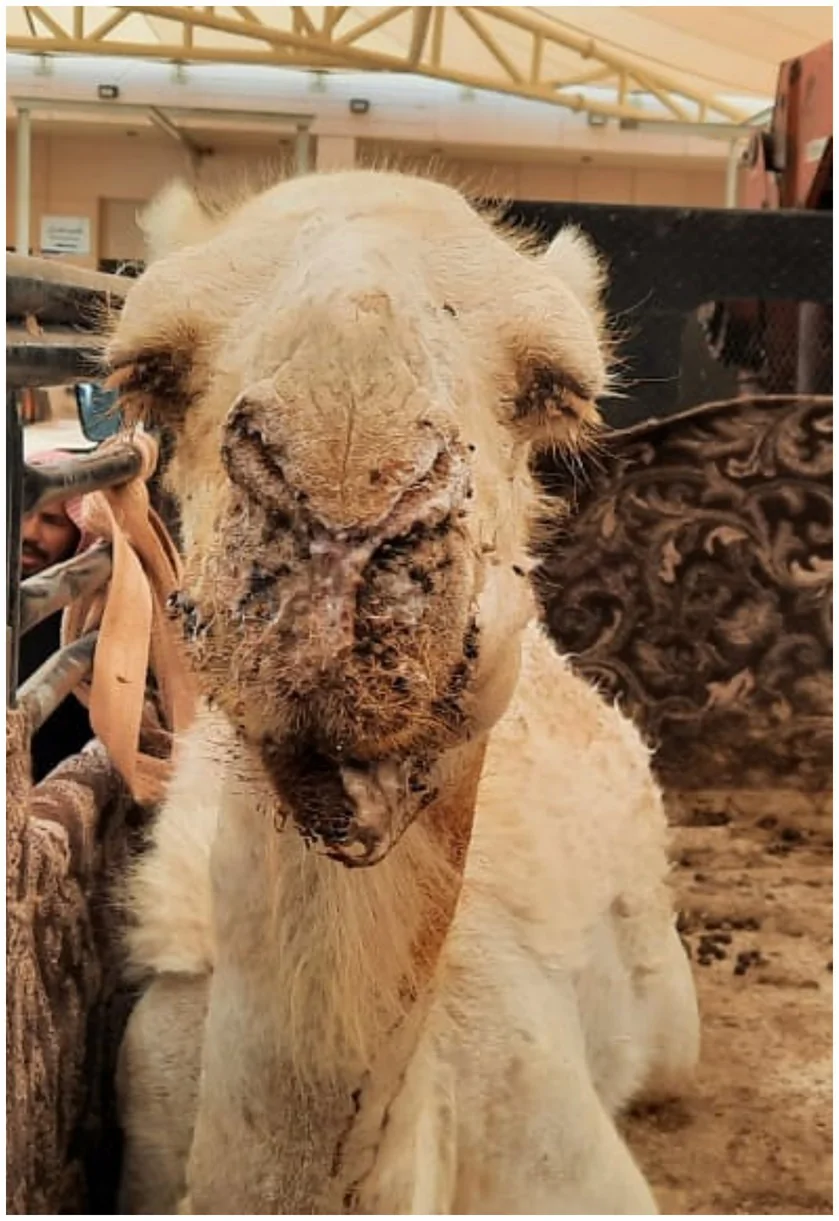
Camel calf, 4-month-old presented with mucopurulent discharges from nostrils and oral cavity due to PPRV infection.

In Iran, reverse transcription polymerase chain reaction (RT-PCR) detected PPRV in the lymph node and lung samples from dead camels, confirming PPRV as the cause of lesions observed in respiratory and gastrointestinal tracts (41). A serological survey of clinically healthy slaughtered camels of various ages and both sexes using ELISA test further demonstrated exposure to PPRV (50). In Saudi Arabia, PPRV-specific antibodies were detected in 2.92% of dromedary camel sera from eastern and southern regions. These findings indicate that camels are exposed to PPRV infection and is a fundamental host in maintenance and spread of the virus among small ruminants across the Arabian Peninsula (45). A recent report on the seropositivity of PPR antibodies in 225 camels tested using an ELISA in the Al-Ahsa region of KSA indicated a percentage of 2.7% (6 out of 225) (51). Overall, these findings highlight the epidemiological significance of PPRV in camels and underscore the need for enhanced surveillance, further research, and integrated control strategies to better understand and mitigate their role in the transmission and persistence of the disease (Figure 1).

### 2.2 Bovine viral diarrhea virus

BVDV belongs to the genus Pesti virus within the Flaviviridae family (52), and is the causative agent of bovine viral diarrhea (BVD) disease. The pathogenesis and epidemiology of BVDV in cattle and camels are influenced by the two genotypes of the virus, BVDV-1 and BVDV-2, each of which has cytopathic and noncytopathic biotypes (53). BVDV was observed with higher frequency in younger animals and aborted dams (54).

The serological prevalence of BVDV in camels had extensively studied. Camels (348 sera) were tested for antibodies against BVDV in Miesso district, Ethiopia, among which eight cases were positive (2.29%). In younger camels, the seroprevalence of BVDV was higher (6.82%) compared to adults (1.64%). Similarly, camels with a history of abortion had a higher seroprevalence (2.38%) than those without any history of abortion (0.00%). Seroprevalence did not differ significantly across parities (54). In southern Egypt, two out of 92 (2.2%) camels were positive for BVDV, indicating the virus is emerging in camels (55). Tesfaye et al. (54) described BVD as primarily a cattle disease but one that is emerging in camels. In Iraq, sera from dromedary camels were collected from areas surrounding Baghdad; a 12 out of 88 (13.63%) were positive for BVDV antibodies (56).

**Figure 2:**
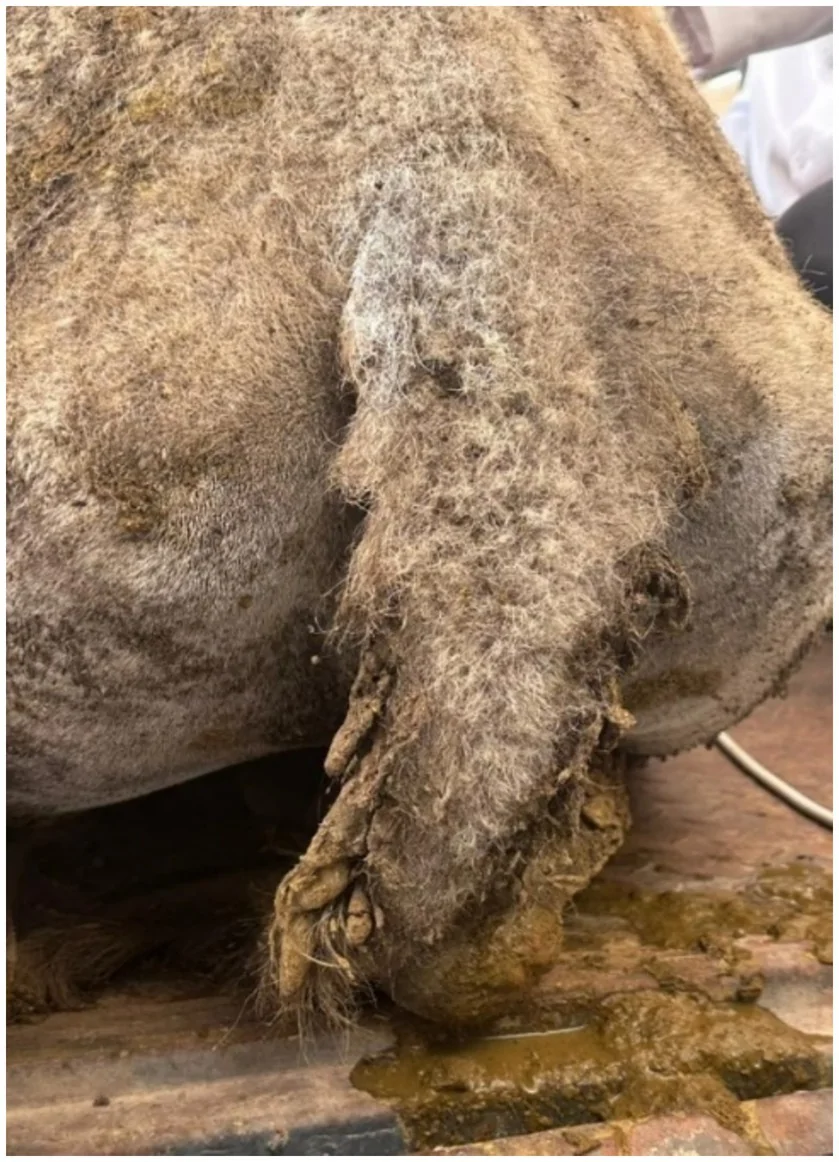
Adult female camel (8-year-old) submitted to Qassim University Veterinary Hospital suffering clinically from severe chronic diarrhea, emaciation, harsh hair, and fever for several weeks postabortion implicating BVDV infection.

BVDV is an international concern which culminates in major economic losses since it causes abortion, lowers the rate of conception and milk production, and lengthens the time between calvings. Additionally, the disease causes respiratory disorders, diarrhea, and reproductive failure in camels (57). BVDV infection in cattle reduces the growth rates, decreases the milk yield, and induces the reproductive failure, and mortality. Infection manifests clinically as fever, mouth ulcers, anorexia, diarrhea, abortion, poor body condition, and occasionally congenital abnormalities (34, 53). According to Agnew, BVDV infection in camelids causes respiratory and reproductive tract diseases, abortion, birth of persistently infected offspring, stillbirth, newborn death, congenital malformation, and chronic respiratory or gastrointestinal disease. In persistently infected alpacas, viral antigens were widely distributed in neurons, endothelial cells, vascular tunica media myocytes, and macrophages in intestinal submucosa and lymph nodes, suggesting potential for viral transmission and effects on fertility (58) (Figure 2).

The risk factor associated with BVDV infection in dromedary camels, including co-housing with sheep, and goats was studied in abattoir surveillance and semi-closed herds in eastern Saudi Arabia (59). Positive sera for the BVDV antibodies were detected by ELISA in 46/1012 dromedary camels, and in 3/84 goats, but the sheep samples (21) were not positive. Dromedary camels and goats in this study were not vaccinated against BVDV, indicating natural exposure to BVDV. These results suggest that camels and goats may play a role in BVDV transmission even in the absence of clinical signs (59). Keeping dromedary camels in mixed herds with sheep and goats may represent an important source of BVDV infection for all ruminants species (60). Camels (111 serum samples) living in mixed herds with sheep and goats in Algeria showed a positive serum rate of 9.0% for the specific BVDV antibodies, while 41.4% of cases are tested positive for BVDV antigen, indicating active viral circulation (60). In Turkey, BVDV-specific antibodies were detected in 16.8% of camel serum samples, suggesting previous exposure, whereas none tested positive for viral antigen, indicating no active infection (61). In another study of 92 camels, seropositivity rates of 32.61, 2.17, 58.7, and 21.74% were reported for Bovine enterovirus-1, Bovine herpesvirus-1, BVDV, and, Parainfluenza-3 (PI-3), respectively, showing that multiple viral infections are common in Western Turkey (62). In Egypt, a study assessing the role of camels in BVDV transmission to cattle examined 50 camels and 50 cattle clinically, serological, virologically, and molecularly twice at one-month intervals (63). Clinical examination revealed no signs of BVDV in camels, even in animals positive by ELISA, virus isolation and RT-PCR. All camels were ELISA-negative at both time points except two, which were weakly positive during the second sampling, while 16/50 camels were positive by virus isolation and RT-PCR. These findings indicate that camels can become BVDV positive shortly after exposure and remain infectious for extended periods without clinical signs (63).

In Iran, camel sera (137 cases) collected from Khorein abattoir near Tehran were tested for presence of BVDV neutralizing antibodies. Camel sera of 27 cases (19.7%) were positive for BVDV by using serum neutralization test, a rate substantially higher than that reported in many other countries (64). In Saudi Arabia, BVDV is known to cause reproductive disorders in camels, particularly leading to uterine infections and abortions. The overall seroprevalence of BVDV was reported to be 29.1% in 182 sera collected from adult female dromedary camels with a history of reproductive disorders including uterine infection and abortion in central, northern and eastern regions (65). Growing evidence indicates that BVDV is increasingly reported in camels, with highly variable seroprevalence and predominantly subclinical infections. This supports the view that camels may function as silent reservoirs, underscoring the need for enhanced surveillance and more effective control measures in mixed-species production systems.

### 2.3 Coronaviruses

Coronaviruses are enclosed RNA viruses with clinical severity ranging from asymptomatic to fatal that can cause respiratory, intestinal, or systemic infections in a range of mammalian hosts. The coronaviral spike protein, which promotes the union of the viral and host cell membranes to start cellular infection, essentially determines the host range and tissue tropism (66). A severe respiratory illness with a high case-fatality rate in humans is caused by the Middle East respiratory syndrome coronavirus (MERS-CoV). Since MERS-CoV first appeared in the middle of 2012, the World Health Organization has reported 2,578 laboratory-confirmed human cases in 27 countries, resulting in 888 known fatalities from the infection and its consequences. The primary reservoir host of MERS-CoV is thought to be dromedary camels, which can infect humans zoonotically (67). Hemida et al. (68) suggested that camels rarely transmit the infection to human after examining 191 persons working in close contact with camels, all of whom were seronegative. On June 13, 2012, the MERS-CoV was discovered in a patient in the KSA who had severe pneumonia and renal failure (69).

A serosurvey of dromedary camels across the middle east and Africa was performed to elucidate the epidemiology of the infection. Among 3,821 samples from seven countries (Egypt, Uganda, Senegal, Tunisia, Jordan, KSA, and Iraq), the overall seroprevalence of MERS-CoV antibodies was 73.7%, with a juvenile (2 years old) seroprevalence rate of 30.3% (70). The earliest evidence of MERS-CoV infection in camels dates back to 1983, when MERS-CoV-specific antibodies were detected in serum samples from Somalia and Sudan, with 81% testing positive (71). The median rate of nasal shedding of MERS-CoV in camels under 2 years old was found to be 34% (718 out of 2,612; range 0–100%) according to virological investigations that included an age breakdown. In contrast, the shedding rate in camels over 2 years old was only 2% (91 out of 1,142; range 0–100%) (72).

The clinical signs in experimentally infected dromedary camels with MERS-CoV include rhinorrhea and mild fever (73–75). On necropsy of at 5 days post-infection, histologic lesions were observed in the pseudostratified epithelial cells of the upper respiratory tract (nasal turbinate, olfactory epithelium, pharynx, and larynx) and lower tract (trachea, bronchi, and bronchioles), but not in the alveoli (73–75). The lesions were described as mild to moderate acute intraepithelial and submucosal inflammation with loss of pseudostratified epithelial cells and multifocal necrosis, which is comparable to human common cold pathogenesis. The epithelium showed mild to moderate infiltration of neutrophils with few numbers of macrophages, and similar cellular infiltration was observed in the submucosa (75). The detection of virus in the upper respiratory tract and trachea, but not in the lungs, may explain the absence of systemic infection among naturally infected camels and the efficient camel-to-camel and camel-to-human transmission (75). In addition, viral antigen has been detected in the tonsils and mediastinal and retropharyncheal lymph nodes of experimentally infected camels (73–75).

To assess natural MERS-coV infection in dromedary camels in Al-Ahsa, Saudi Arabia, nasal swabs and lung tissues were tested with RT-PCR. Out of 96 nasal swab samples collected from live camels, 28 (29.2%) tested positive. In contrast, 56 out of 91 (61.5%) lung tissue samples obtained from carcasses were positive. The prevalence of positive samples was higher in younger animals (those under 4 years of age) compared to adults (those over 4 years of age) (74). Four MERS-CoV–positive calves exhibited mild respiratory signs (cough, sneezing, respiratory discharge), fever, and loss of appetite. A dromedary camel herd sampled during calving season tested positive for MERS-CoV by RT-PCR in both calves and adults (73). Viral isolation was conducted from nasal secretions and fecal matter, with a higher frequency of isolation from nasal samples compared to feces. Hemida et al. (73) suggested that dromedary camels are natural hosts of MERS-CoV rather than passive carriers, which contrasts with the interpretation by Adney et al. Together, these findings indicate that MERS-CoV is highly prevalent among dromedary camels, especially in younger animals, and that camels act as the principal reservoir host, typically exhibiting mild or subclinical infections, thereby underscoring their key role in sustaining, and facilitating zoonotic transmission of the virus.

### 2.4 Parainfluenza 3 virus

Pneumonic lung specimens (281) collected from slaughterhouses in four different areas of Sudan during an outbreak of pneumonic lesions in dromedary camels, six samples (2.1%) were found positive for PIV3 antigen by ELISA, and four specimens yielded the virus by isolation, while two samples were positive by RT-PCR (76). PIV3 was detected via hemagglutination inhibition testing and then also isolated from the imported live camels from Djibouti to Egypt (77) Similarly, PI3V antibodies were detected in sera of local as well as Somali– imported camels in Egypt in a prevalence rate of 27.87% (78). Co-circulation of dromedary camel PIV3 (dcPIV3), a novel species within the genus *Respirovirus,* along with MERS CoV in a dromedary camel herd (nine camel calves) manifesting signs of respiratory tract infection such as clear nasal discharge and fever, has been recently recorded in Emirates and confirmed as a causal pathogen by virus isolation, identification and genomic sequence (79). In Turkey, among 92 camel serum samples tested for neutralization, 20 (21.74%) were seropositive for PIV-3 antibodies (62).

The isolation and molecular identification of PIV3 from camels with respiratory signs indicate its distribution in the southern region of Ethiopia and its involvement in respiratory disease outbreaks in camels (80). In Saudi Arabia, the seroprevalence rates of bovine PI-3 were 39% in cattle distributed in different regions in KSA (81) but not recorded yet in dromedary camels. It is recommended to further investigate the role of bovine PI3V in respiratory manifestations among camel herds n KSA. Human parainfluenza viruses (PIVs) are important yet often neglected pathogens causing lower respiratory tract infection in younger age children (82), similarly PI3V still neglected in veterinary research and scarcity of studies investigating its role in respiratory distress in camel calves. Current evidence indicates that PIV3 and other related respiroviruses circulate in camel populations and may play a role in respiratory illness. This highlights the importance of further epidemiological and molecular research to better understand their significance and impact, particularly in areas where information is still scarce.

### 2.5 Adenovirus

Adenovirus infections, although less common, can occur alongside PPRV in camels, suggesting that co-infection may exacerbate respiratory disease severity (4). Pneumonic lung specimens from Central and Northern Sudan were shown to contain the adenovirus 3 antigen. Three out of 239 pneumonic camel lung tissues tested positive for the adenovirus 3 antigen (1.3%) using sandwich ELISA kits (83). Adenovirus type 3 is recognized as a causative agent of respiratory infections in cattle and has been reported globally. In camelids, adenovirus has historically been considered a non-pathogenic viral infection (30), although Intisar et al. (83) reported that adenovirus-3 antigen caused pathological effects in camel lungs. Adeno and rotaviruses have been implicated solely or in association with enteric microbes in the camel calf diarrhea (84). Nine of 10 pneumonic lung tissue samples were positive for, Respiratory syncytial virus (RSV) and two of six were positive for Adenovirus in a slaughterhouse study in Ethiopia (85). Although adenovirus appears to be relatively uncommon in camels, its identification in pneumonic lung tissues and its association with other pathogens indicate that it may contribute to respiratory and enteric diseases, highlighting the need for further research to better understand its clinical and epidemiological importance.

### 2.6 Respiratory syncytial virus

RSV is a key virus involved in respiratory infections across various animal species (86). It belongs to the genus *Pneumovirus* of the family *Paramyxoviridae*, subfamily *Pneumoviridae*, and is the cause of respiratory syncytial disease (87). RSV is a delicate virus primarily transmitted between animals through aerosolized secretions (88). The abrupt onset of pyrexia, deep breathing, lethargy, rhinitis, nasal discharge, and cough are symptoms of RSV infection in calves (89). After necropsy, bronchiolitis and interstitial pneumonia with edema and emphysema affecting all lung lobes, which may progress to bronchopneumonia and death were the main pathological findings (89). Secondary *Pasteurella haemolytica* infection is common, and high morbidity and low mortality are typically linked to the RSV disease (90). The incidence of RSV infections, which are more common in the fall and winter, may be influenced by environmental factors. Besides, climatic conditions that favoring viral spread, seasonal herd management practices such as cattle indoors housing and mixing might contribute to increased seasonal incidence of RSV infection (88).

In Sudan, Abbas et al. (91) reported that respiratory diseases in camels were complained by 28.6% of pastoralists in Eastern Sudan. PI3V, influenza virus A and B, adenovirus, RSV and infectious bovine rhinotracheitis (IBR) are encountered in respiratory infections in camels (92). In apparently healthy camels in Nigeria, RSV antibodies were detected by complement fixation test in 0.6% of 157 serum samples of slaughtered camels (93). In Egypt, also, 9.8% of 580 apparently healthy camel sera tested were positive for RSV antibodies (94). In the other hand, RSV detection was linked to pneumonic lesions in camels in several studies. RSV antigen was detected in 4 (1.4%) out of 280 pneumonic lungs of dromedary camels, either in outbreaks or slaughterhouse specimens, all originating from Central Sudan (95). One lung showing interstitial pneumonia out of 45 camel lungs of different ages and localities in Sudan tested positive for a mixed infection, including PIV3, adenovirus, RSV and BVDV. No bacteria were isolated from the affected lung; however, four type of viral antibodies were detected using the fluorescence antibody technique (96). While RSV appears to be relatively uncommon in camels, its presence in respiratory lesions and its frequent association with other viral infections suggest it may contribute to complex, multifactorial respiratory disease conditions, underscoring the need for further research into its epidemiology and overall impact in camel populations. Table 1 summarizes the reported viruses causing pneumo−/ enteritis in camels in Saudi Arabia.

**Table 1:** Summary of the reported viruses causing pneumo−/ enteritis in camels in Saudi Arabia.

Viral pathogen	Type of sample	Study population	Diagnostic method	No. of samples	No. of positive	Reference
PPRV	Serum	camel survey in contact with small ruminant	ELISA	461	0	(46)
PPRV	Serum	Camel survey population	ELISA	370	11	(45)
PPRV	blood, nasal and ocular swabs	experimental work pneumonic camels	Virus Isolation, RT-PCR	50	17	(44)
PPRV	Serum	Herd camels	ELISA	225	6	(51)
BVDV	Serum	abattoir camels and semi-closed herd camels	ELISA	1,012	46	(59)
BVDV	Serum	Herd camels with reproductive disorders	ELISA	182	53	(65)
MERS-CoV	Serum	Healthy camel surveillance	ELISA,	222	181	(70)
MERS-CoV	Nasal swab	Healthy camel surveillance	RT-PCR	224	7	(70)
MERS-CoV	Nasal swab	Herd camel surveillance	RT-PCR	96	28	(74)
MERS-CoV	Lung tissue	slaughtered camels	RT-PCR	91	56	(74)
Rotavirus	Serum	Diarrhoetic camels	ELISA	184	27	(35)

### 2.7 Mixed viral infections contributing to pneumo−/enteritis in dromedary camels

Although PPRV, BVDV, and MERS-CoV have traditionally been discussed as independent viral threats in camel populations, available evidence indicates that these viruses may circulate concurrently within the same herd environments, particularly under intensive contact, mixed-species grazing, and high-mobility pastoral systems (4, 5). PPRV and BVDV primarily contribute to respiratory, enteric, and systemic disease through immunosuppression and mucosal damage, potentially increasing susceptibility to secondary bacterial infections such as *P. multocida* (90), whereas MERS-CoV is predominantly associated with respiratory tract involvement and zoonotic transmission risk. Other coronaviruses affect intestine causing enteritis and severe diarrhea in Camelidae (66). Comparative studies suggest that differences in host range, transmission pathways, and tissue tropism influence their epidemiological patterns; however, overlapping circulation may complicate clinical diagnosis because similar respiratory and systemic signs can result from single or multiple viral infections. Mixed infection of (PPRV) and other respiratory viruses including PIV-3, RSV, BHV-1, BVDV, and adenoviruses (4, 62, 96) should be considered within the broader viral ecology of camel herds. These viruses share overlapping epidemiological settings characterized by young-animal susceptibility, close animal contact, herd aggregation, transportation, and environmental stress. PI3V and RSV are principally associated with respiratory tract disease and may compromise mucociliary and epithelial defenses, whereas adenoviruses may contribute to respiratory and systemic disease depending on the virus and host context (76, 77, 97). The limited availability of longitudinal field investigations evaluating simultaneous viral detection highlights an important knowledge gap regarding viral co-infection frequency, interactions between viral agents, and their contribution to severe pneumo-enteritis outcomes in camel calves. Future surveillance programs should therefore incorporate multiplex molecular diagnostics to quantify viral co-circulation and clarify the role of mixed viral infections in camel herd morbidity and mortality. Table 2 summarizes the viral pathogens associated with camel pneumo−/enteritis and their major transmission drivers and potential interaction with pneumo-enteritis.

**Table 2:** Viral pathogens associated with camel pneumo−/enteritis and their predilection, major transmission drivers and potential interaction.

Viral agent	Main camel-associated syndrome	Major transmission drivers	Potential interaction with pneumo-enteritis
PPRV	Respiratory, enteric, systemic disease	Close contact, mixed species herding, herd movement	Immune suppression and mucosal injury facilitating secondary infections
BVDV	Respiratory-enteric disease, reproductive effects	Contact with infected ruminants, mixed livestock systems	Increased susceptibility to bacterial respiratory/intestinal complications
MERS-CoV	Predominantly respiratory disease, zoonotic concern	Camel-to-camel and camel-to-human transmission	May contribute to respiratory compromise and complicate differential diagnosis
PI3V	Respiratory disease	Close contact, mixed species herding, herd movement	Increased susceptibility to bacterial respiratory complications
RSV	Respiratory disease	Close contact, mixed species herding, herd movement	Increased susceptibility to bacterial respiratory complications
Adenovirus	Respiratory disease and potentially systemic tissues	Close contact, mixed species herding, herd movement	Immune suppression and mucosal injury facilitating secondary infections

### 2.8 Zoonotic significance of viral pathogens in camel pneumo-enteritis

The zoonotic diseases transmitted from camels has been reviewed recently by Khalafalla (98) and found that brucellosis, MERS-CoV, *Echinococcus granulosus*, and Rift Valley Fever (RVF) were the subjects of the majority of papers on zoonotic camel diseases (65%) (98, 99). Among viral pathogens associated with camel respiratory disease, MERS-CoV represents the most important zoonotic concern due to its established ability to transmit between dromedary camels and humans (100). Infected camels may serve as reservoirs for viral circulation, with exposure risks particularly relevant for herders, veterinarians, slaughterhouse workers, and individuals involved in close camel contact (100, 101). Although other camel-associated respiratory viruses may primarily affect animal health, their contribution to respiratory disease complexes and potential interactions with emerging zoonotic viruses require continued surveillance. Integrated monitoring of respiratory viruses in camel herds, combined with occupational risk assessment and molecular characterization of circulating strains, is essential for reducing zoonotic spillover risks in camel-producing regions. Table 3 summarized the regional prevalence, host age predilection, clinical presentation, main pathological lesions, and zoonotic risk levels of viral pathogens associated with pneumo−/enteritis in camels.

**Table 3:** The regional prevalence, host age predilection, clinical presentation, main pathological lesions, and zoonotic risk levels of viral pathogens associated with pneumo−/enteritis in camels.

Viral pathogen	Regional prevalence/occurrence	Host age predilection	Primary clinical syndrome	Main clinical lesions	Zoonotic risk level
PPRV	Reported in camel populations in Middle Eastern, African, and Asian camel-producing regions; outbreaks associated with mixed-species herding	Mainly young and immunologically naïve camel calves	Fever, respiratory distress, nasal discharge, diarrhea, depression	Bronchointerstitial pneumonia, lymphoid depletion, intestinal mucosal inflammation, enteritis	Low (primarily animal pathogen; public health significance limited)
BVDV	Detected in camel herds in regions with contact between camels and cattle/small ruminants	Young calves more susceptible due to immature immunity	Respiratory–enteric disease, diarrhea, poor growth, immune suppression	Respiratory mucosal injury, enteritis, lymphoid tissue changes	Low (not considered an established camel-to-human zoonosis)
MERS-CoV	Widely reported in dromedary camel populations across Saudi Arabia and other Arabian Peninsula countries	All ages can be infected; higher epidemiological importance in adult reservoir camels	Respiratory infection, nasal discharge, coughing; potential subclinical carriage	Respiratory epithelial damage, airway inflammation, pulmonary lesions	High (recognized zoonotic pathogen with camel-to-human transmission)
Other camel-associated respiratory viruses (PI3V, RSV, adenovirus)	Reported variably depending on surveillance intensity and molecular testing availability	Primarily young and stressed animals	Respiratory disease, often complicated by secondary bacterial infection	Bronchitis, pneumonia, mucosal inflammation	Low to uncertain

## 3 Overview of bacterial pathogens contributing pneumo−/enteritis in dromedary camels

### 3.1 Pasteurella

*Pasteurella multocida*, the infectious agent of fowl cholera first identified by Louis Pasteur in 1881, a Gram-negative bacterium belonging to the family *Pasteurellaceae*. *P. multocida* is the causative agent of many diseases in a wide range of wild and domestic animal hosts, and infections that affect livestock and lead to significant economic losses worldwide (102). *P*. *multocida* is part of the normal flora of the camel respiratory tract, but it becomes pathogenic when the camel’s resistance is diminished by adverse ecological conditions (103). *P. multocida* causes hemorrhagic septicemia (HS) or pasteurellosis in camels worldwide (104, 105).

*P. multocida* is a common respiratory flora in camels, but it can become pathogenic when detrimental environmental factors like abrupt weather changes, long-distance transportation, malnutrition, viral infections, and severe parasite infestations like trypanosomiasis reduce the camel host’s resistance (97, 106). Under these conditions, *P. multocida* can invade the host tissues and cause a disease. *P. multocida* may induce septicemia in camels within 24 h, reflected clinically by pyrexia, swelling of the throat, pulmonary edema, fibrinous pneumonia, and diarrhea, with death typically occurring within 2–3 days (107). *P. multocida* (capsular type E) infections were detected in camels in the Kenyan counties. To reduce the spread of hemorrhagic septicemia, it is crucial to implement proper nutrition, treatment, and vaccination, as well as to prevent herding camels in congested areas, especially in humid weather (103).

*P. multocida* is the primary causative agent of hemorrhagic septicemia in camels and detected in five cases of 150 camels (3.3%) with respiratory distress through bacterial isolation and identification from 30 nasal swabs and 120 lung tissues (108). The first clinical signs of hemorrhagic septicemia in cattle and buffaloes include fever, dullness, and reluctance to move. Salivation and a serous nasal discharge develop as the disease worsens. Edematous swellings first appear in the pharynx and can spread to the brisket and ventral cervical region. As the affection progresses, the mucous membranes get congested, and respiratory difficulties may arise. (37, 109, 110). Peracute and acute forms of pasteurellosis, have been documented in association with *P. multocida* infection in dromedary camels. Fever, mucopurulent nasal secretions, lacrimation, tachycardia, mucosal congestion, pneumonia, edema of the neck, throat, and related lymph nodes, and occasionally the hindquarters are among the clinical symptoms in camels (37, 110, 111). Ayoub et al. (110) documented an outbreak of acute pneumonic pasteurellosis among camels reared under Cholistan desert conditions, where naturally infected animals showed severe respiratory disease and characteristic pulmonary lesions associated with *P. multocida* infection. Notably, the affected camels also exhibited enteric and systemic pathological changes, including severe enteritis, pericarditis, and inflammatory lesions in multiple organs, supporting the concept that respiratory infections in camels may occur as part of broader multisystem disease complexes rather than isolated pulmonary disorders. These findings provide regional evidence for incorporating bacterial pneumonia outbreaks into the understanding of camel pneumo-enteritis-associated morbidity and mortality in arid pastoral production systems (110).

**Figure 3:**
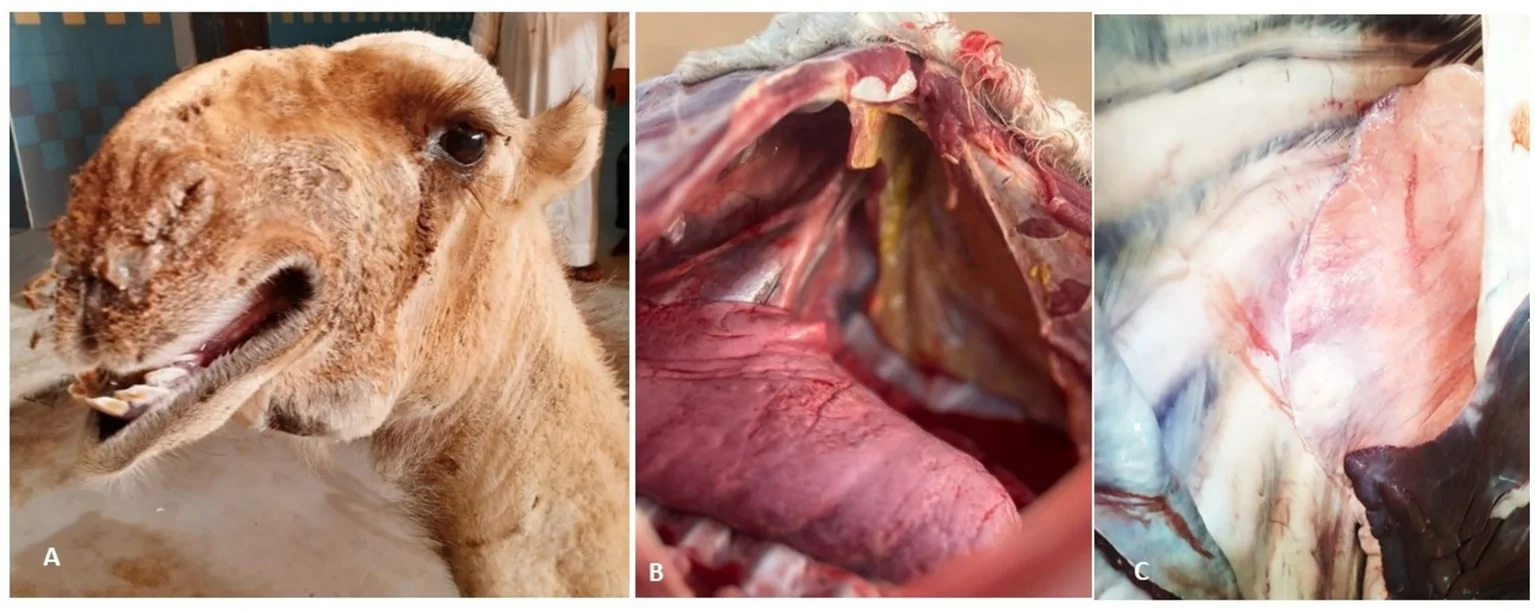
**(A)** Camel calves (5-month-old) with mucopurulent discharges from nares and severe lacrimation, swelling of throat, and regional lymph nodes. **(B)** Necropsy of dead camel calf (6-month-old) revealed accumulation of serosanguineous exudate in the pleural cavity and fibrinous pleuropneumonia in camel calves and **(C)** Fibrinous adhesion between lungs and parietal pleural of the thoracic cavity due to *P. multocida* infection.

Pyrexia, severe dyspnea with nasal thick gummy material, and abortion during late gestation were among clinical findings reported during an outbreak of hemorrhagic septicemia in camels (112). Post-mortem examination of febrile carcasses revealed generalized visceral congestion, serosal petechial hemorrhages, accumulation of serosanguineous exudate in the pleural and peritoneal cavities, and pneumonia. *P. multocida* subsp. *multocida* was isolated from both clinical and morbid cases of affected camels (112). Finally, Concurrent or predisposed viral or bacterial ailments, as well as environmental and stress-related factors like transportation, co-mingling, and crowding, are linked to *P. multocida*-induced pneumonia (111, 113). The high temperature and harsh environment in grazing camels may affect the electrolyte and trace element balance in camel body as well as increased oxidative stress. It was found that electrolyte profiles, trace element levels and oxidants antioxidants balance are greatly disturbed in camel-calves with pneumonic pasteurellosis in KSA (114). *P. multocida* is an important opportunistic pathogen in camels that, particularly under stress or predisposing conditions, can quickly cause severe respiratory disease and hemorrhagic septicemia. This underscores the need for effective preventive measures, prompt diagnosis, and well-implemented control strategies to reduce its economic impact (Figure 3).

### 3.2 Escherichia coli

Bacterial pathogens are the main causes of diarrhea in camel calves (115). In Saudi Arabia, diarrhea in camel calves is primarily associated with *E. coli* (58.2%) and enterotoxigenic *E. coli* (ETEC) pathotype (7%) infections. *E. coli* is strongly associated with camel calf diarrhea (35). Microbial identification of fecal samples from diarrheic camel calves resulted in 66% (69/121), and 81% (81/100) were positive for *E. coli* in Sudan (116). In another study, *E. coli* isolates were recovered from 52/190 (27.3%) diarrheic specimens obtained from young camels in KSA (115). *E. coli* septicemia has been detected in young camelids (*Camelus dromedarius*) aged between 2 and 4 weeks (30, 117, 118). The morbidity and mortality rates reported in dromedary calves infected with *E. coli* were 30 and 100%, respectively (119). Indigestion caused by overfeeding can lead to intestinal stasis, which may be exacerbated by toxins produced by bacteria like *E. coli* or *C. perfringens*, resulting in severe abdominal colic and obstruction (120).

**Figure 4:**
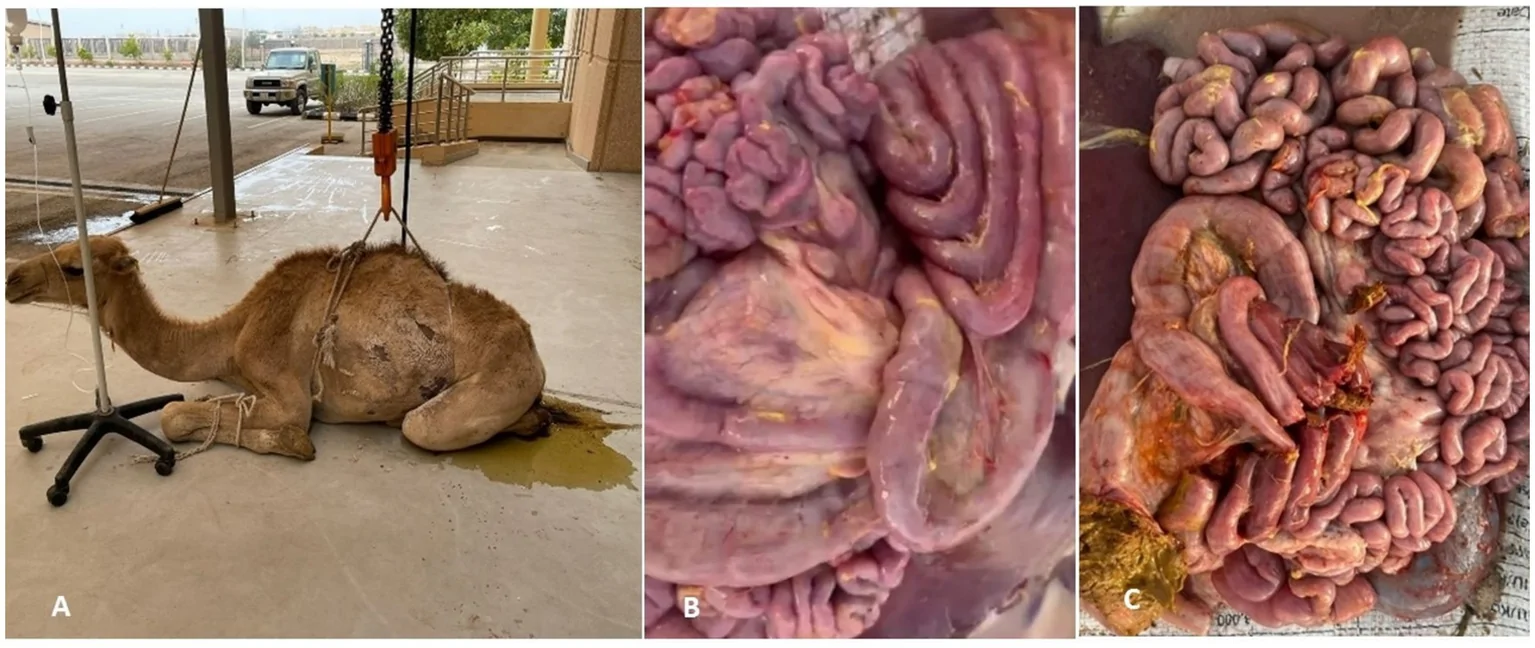
**(A)** Camel calf (3-month-old) presented with a greenish watery diarrhoea due to *E. coli* infection. **(B,C)** Necropsy of a dead suckling camel calf (2-week-old) revealed impaction of the intestine with pasty food content inducing function obstruction of the intestine and eventually death due to *E. coli* infection. **(C)** Opened intestine revealed inspissated food material.

In camel calves, intestinal issues such as impaction are often linked to management practices, including insufficient or excessive intake of colostrum and milk (121). Early-life immune protection plays a critical role in determining susceptibility to respiratory and enteric infections in camel calves. Camel milk contains several immunologically active components, including immunoglobulins, lactoferrin, lactoperoxidase, and cytokines, which contribute to mucosal defense and regulation of inflammatory responses during the neonatal period. Protective proteins and cytokine profiles in camel milk and serum during the first 2 months postpartum demonstrated dynamic changes in immune mediators, highlighting the importance of maternal immune factors in calf health and disease resistance (31). Disruption of these protective mechanisms, whether through inadequate colostrum intake, maternal immune variation, or infectious stressors, may reduce mucosal barrier function and increase vulnerability to viral infections followed by secondary bacterial complications in camel pneumo-enteritis. Therefore, characterization of camel milk-derived immune biomarkers may provide valuable indicators for predicting calf susceptibility and improving early intervention strategies in affected herds (32) (Figure 4).

Many *E. coli* pathotypes, such as enterotoxigenic (ETEC), Shiga toxin producing (VTEC or STEC), necrotoxigenic (NTEC), enteropathogenic (EPEC), enterohemorrhagic (EHEC), enteroaggregative (EAggEC), and enteroinvasive (EIEC) strains, are frequently responsible for diarrhea and/ or enteritis in both humans and farm animals (122). Shiga toxin-producing *E. coli* (STEC) has been isolated from fecal samples of African dromedary camels in Kenya (123). In contrast, El-Sayed et al. found that camels have no epidemiological significance in STEC infection and transmission in samples from five African countries.

Hemolytic uremic syndrome and enterohemorrhagic colitis can be driven on by *E. coli O157: H7* (124). Enteropathogenic *E. coli* serotypes were isolated from diarrheic neonatal camel calves (17.1%) in Egypt, and these isolates exhibited virulence-associated and multiple drug resistance genes (125). In KSA, camels are implicated as a potential reservoir for extended-spectrum *β*-lactamase-producing *E. coli* and are suspected of aggravating human infections (126). Fadlelmula et al. reported that camels may act as a reservoir for *E. coli* strains that are resistant, adding to the growing problem of antibiotic resistance in Saudi Arabia. They verified the presence of multidrug-resistant strains, such as the *E. coli* ST131 clone, in both humans and camels and documented a high prevalence rate of ESBL-producing *E. coli* (ESBL-EC) in camels for the first time in KSA. *E. coli* was isolated from camel intestines (26.0%) and human urinary tract infections (33.0%). ESBL-EC *E. coli* was found in 26.9% (*n* = 5/26) of camel intestinal and 36.4% (*n* = 8/33) of human urinary samples. For camels and human samples, the multidrug resistance index was 0.13 and 0.17, respectively. Drug-resistant *E. coli* (O25b: H4-ST131 clone) were found in both human and camel samples, providing more evidence that camels may serve as a reservoir for resistant *E. coli* strains in Saudi Arabia (126). Antimicrobial resistance and molecular characterization of virulence genes of *E. coli* strains isolated from healthy (62 cases) and diarrhea-suffering camel calves (58 cases) in Tunisia showed that the strains primarily belonged to phylogenetic group B1, which accounted for 52.9 and 52.8% of the strains in healthy herds and diarrhea-suffering herds, respectively. The highest frequency of antibiotic resistance were against tetracycline and ampicillin (52.8 and 37.1%, respectively) (127). Multidrug resistant *E. coli* was isolated from tissues of dead adult racing dromedary camels suffering from coli septicemia (128). Additionally, *E. coli* has been isolated from reproductive disorders (129), renal abscesses and pyelonephritis (130), and pneumonia in camels (131). In Saudi Arabia, *E. coli* O157: H7 was detected in healthy camel feces collected from slaughterhouse at a prevalence of 2.4%, with no significant difference compared with sheep and goats (*p* > 0.05) (124). *Escherichia coli* is a key enteric pathogen in camel calves, playing a major role in diarrhea, septicemia, and elevated mortality. Its wide range of pathogenic strains, along with rising antimicrobial resistance, underscores the importance of improved management practices, better hygiene, and targeted control measures to minimize its impact on both animal and public health.

### 3.3 Clostridium perfringens

*Clostridium perfringens,* a Gram-positive, anaerobic, spore-forming bacterium, is responsible for a wide range of histotoxic and enterotoxic diseases affecting both humans and animals (132). *C. perfringens* type A is the most prevalent strain of *C. perfringens* found in camels and humans in Egypt (133). Similar findings have also been reported in Saudi Arabia, where this strain was identified in cattle, sheep, camels, and goats (134). *C. perfringens* causes various gastrointestinal infections in most mammalian species, collectively referred to as enterotoxaemia. The bacterium can also cause gas gangrene or malignant edema after infection of the skin and soft tissue (135). Large amounts of toxins produced by *C. perfringens* are absorbed and induce enterotoxaemia. Enterotoxemia outbreaks in dromedary camels are proved to be caused by *C. perfringens* types A, B, and D (135). Dromedary camels usually have clinical signs of diarrhea, recumbency, opisthotonos, and quick death. The identification of *C. perfringens* toxins in the intestinal contents of recently deceased animals provides the basis for the definitive diagnosis of enterotoxemia (136). Toxoid vaccinations have been demonstrated to protect camels against enterotoxemia, and intravenous infusion of bovine *C. perfringens* hyperimmune serum is utilized for treatment to quickly neutralize circulating toxins (135).

*Clostridium perfringens* is an environmentally ubiquitous bacterium that frequently colonizes the intestinal tract of healthy humans and animals as part of the normal gut flora (136), so the diagnostic challenge in enterotoxemia is the detection of toxin or toxin-encoding genes. The prevalence of *C. perfringens* was lowest in cattle (23.1% in healthy animals and 14.7% in diseased animals), and greatest in camels (20% in healthy animals and 25% in diseased camels). Real-time quantitative PCR (rt-qPCR) and ELISA yielded inconsistent results for the key toxin-encoding genes, cpa, etx, and cpb. The Delta region of Egypt was shown to have a high prevalence of *C. perfringens* type A and D infection, especially in camels (136). In KSA, *C. perfringens* was isolated and genotyped from both diarrheic and healthy dromedary camels, as well as from pastures and herders. A total of 262 out of 465 isolates (56.3%) were successfully recovered. *C. perfringens* type A isolates expressed *cpa^+^* and *cpb2*^+^ genes, whereas the *cpe* and *iap* genes were not detected among the isolated strains (137). *C. perfringens* type A with the *cpa+* gene was the most prevalent toxin type. Most of the isolates were resistant to at least one of the 10 antimicrobials, proving the emergence of multiple-drug-resistant *C. perfringens* (137). The incidence of sudden death in camels reached 15% due to clostridial enterotoxemia outbreaks in Saudi Arabia. *C. perfringens* enterotoxins identified in the rumen, intestinal contents, and intestinal wall, indicating their role in disease pathogenesis and fatal cases (36).

*C. perfringens* enterotoxaemia has been recognized as a cause of sudden death in camels, and sudden dietary changes, particularly the introduction of high-concentrate rations (≈18%), can disrupt the gut microbiome, leading to *C. perfringens* proliferation and the yielding of potent toxins (36). Vaccination with a polyvalent clostridial antiserum of bovine origin administered intravenously to camels, as well as the absence of vaccination and the presence of secondary infections like acute *Trypanosoma evansi* infection and salmonellosis, predispose camels to enterotoxemia outbreaks (18, 138). The analysis of genetic, biochemical, and microbial factors linked to diarrhea in camel calves identified several key pathogens, including *Salmonella* spp.*, Clostridium perfringens, Proteus* spp., and *E. coli*. These bacteria possess distinct virulence genes, such as *papC, eaeA, iutA, hilA, sopB, plc*, and *ureC* (14).

**Figure 5:**
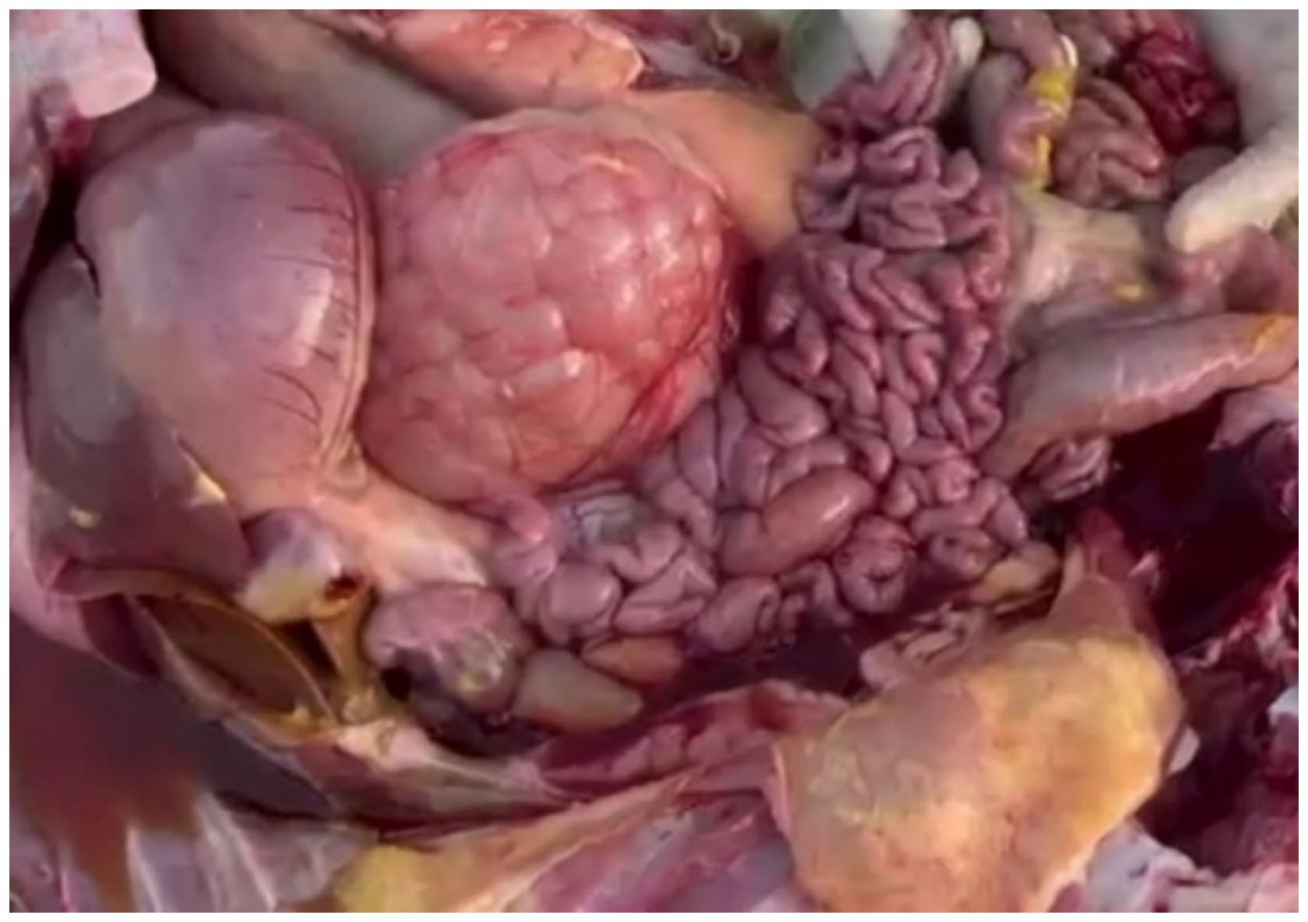
Necropsy of dead camel calf (4-month-old) revealed the intestine appeared hemorrhagic, congested serosal blood vessels and luminal gas bubbles due to enterotoxemia.

Necropsy findings in camels died during enterotoxaemia outbreaks in Pakistan revealed petechiation on the serosal surfaces and congestion of visceral organs, including kidneys, spleen, small and large intestines, heart, and lungs (139). Multiplex PCR analysis indicated the presence of *C. perfringens* type A possessing *α* and β2 toxin genes, and type D possessing the epsilon toxin gene. Notably, mortality rates were significantly higher in cases linked to *C. perfringens* type D compared to those associated with type A (139). *Clostridium perfringens* is a significant enteric pathogen in camels, capable of causing rapidly fatal enterotoxemia under favorable predisposing conditions. Its widespread distribution, diversity of toxins, and rising antimicrobial resistance highlight the need for vaccination, sound management practices, and timely diagnostic measures (Figure 5).

### 3.4 Mycobacterium bovis

Tuberculosis is a chronic granulomatous zoonosis caused by *M. tuberculosis* complex that affects various animal species, including camels (140). An epidemiological investigation revealed a 1.7% (184/10,903) prevalence of suspected tuberculous lesions in apparently healthy camels with gross tuberculous lesions examined at slaughterhouses in delta, Egypt. The suspected cases were further examined with ELISA, of which 124/184 (67.39%) sera were positive. Molecular characterization of the bacterial culture obtained from tuberculous lesions further confirmed *M. bovis* infection in camels (140). Transmission of *M. bovis* between animals occurs mainly via aerosols, as well as through direct contact, shared feed and water sources, and suckling (141). *Mycobacterium* was isolated from tuberculous lesions in the lung (9.4%, 3/32) and lymph nodes (2.4%, 1/41) from apparently healthy slaughtered camels examined at the Cairo abattoir. The isolated strains exhibited characteristic cultural and phenotypic features of the genus *Mycobacterium*. Further identification using Sanger sequencing identified the strain as *M. tuberculosis* variant *Mycobacterium bovis,* designated DRC-EG-CAMEL (PQ036932) (142).

In a five-year study, the pathological characters of tuberculosis in naturally infected dromedary camels caused by *Mycobacterium bovis* were classified based on occurrence and distribution of gross TB lesions, into two forms: pulmonary TB (*n* = 15) and disseminated TB (*n* = 3) form (143). Giant cells and acid-fast bacilli, primary components of the typical granulomatous lesions, are observed in the disseminated form, and occasionally in the pulmonary form. Tuberculosis detection was varied according to diagnostic method; 10 (55.55%) by the single intradermal tuberculin test (SIDT), 13 (72.22%) by the Ziehl-Neelsen (ZN) stain, and 18 (100%) by the IS6110 PCR of tissue lesions from infected camels (143). Predominance of pulmonary TB in camels, indicating inhalation as the main way of exposure in camel herds (143). Tuberculous lesions caused by *M. caprae* (SB0157 spoligotype) is of primary chronic and generalized type was recorded in one camel, and the lesions were mainly distributed in the abdominal organs. Various nodular lesions of different sizes (0.5–3 cm of diameter) were detected in the spleen, liver, and pulmonary parenchyma. The lesions revealed calcified caseated center with a multifocal to coalescent whitish, hard, and rough appearance. Swollen mesenteric and mediastinal lymph nodes, as well as miliary involvement of serosal membranes, were recorded (144).

In the UK, which has a well-established camelid sector, an increase in tuberculosis cases has been reported in certain regions, particularly among New World camelids (NWCs). This observation emphasizes the epidemiological significance of mycobacterial infections within camelids and highlights their potential relevance to Old World camelids, including camels, as susceptible hosts (145). A necropsy, along with the observation of typical gross lesions, histopathological pathognomonic lesions, and bacterial culture, can provide a definitive diagnosis of tuberculosis. The lungs, thoracic lymph nodes, as well as liver, spleen, kidney, trachea, and pericardium are among the organs where nodules typically form. Histologically, tuberculous lesions are characterized by the formation of granulomas, which feature central caseous necrosis and variable calcification (142, 145). No reports have been published on tuberculosis in camels in KSA. This lack of documentation may be attributed to the hot and dry climate. Additional investigation into mycobacteriosis in camels is necessary. Although tuberculosis in camels appears to have a relatively low prevalence, the confirmed detection of *Mycobacterium bovis* and other members of the *M. tuberculosis* complex highlights its zoonotic risk and underscores the importance of improved surveillance, diagnostic capacity, and control measures to better understand and reduce its impact.

### 3.5 Streptococcus pyogenes

In a bacteriological study conducted on slaughtered camels with respiratory affections, *Staphylococcus aureus* and *Streptococcus pyogenes* were successfully isolated from nasal swabs and samples of the lungs, bronchial lymph nodes and pleural fluids, indicating their role as important bacterial pathogens associated with respiratory infections in camels (12). *Staphylococcus aureus, Corynebacterium pyogenes, Streptococcus pyogenes, Escherichia coli, Klebsiella pneumonia, Pseudomonas aeruginosa, Trueperella* (formerly *Arcanobacterium*) *pyogenes, Mannheimia haemolytica and Pasteurella multocida* were the most frequently isolated bacterial species from pneumonic camels lesions (10). These findings demonstrate that a diverse range of bacterial pathogens, including *Staphylococcus aureus* and *Streptococcus pyogenes*, are commonly associated with respiratory infections in camels, highlighting the multifactorial nature of camel respiratory disease and the importance of comprehensive diagnostic and control approaches.

### 3.6 *Salmonella* spp

Salmonellosis is an animal disease with significant zoonotic potential that is characterized by intestinal or enterohepatic manifestations. Neonatal scours, abortion, orchitis, pneumonia, and septicemia are among the clinical indications of salmonellosis. (146). Camels may serve as a reservoir host for *Salmonella enteritidis*, according to the identification of the organism in the feces and mesenteric lymph nodes of clinically healthy camels (147). In Ethiopian apparently healthy slaughtered camels, 16 different serotypes of *Salmonella* spp. were identified, including: *Salmonella saintpaul* (38.8%), *S. braenderup* (22.4%), *S. muenchen* (8.6%), *S. kottbus* (6.0%) and *S. havana* (5.2%). *S. typhimurium*, *S. heidelberg* and *S. enteritidis* were also detected (148). Salmonella has been isolated from diarrheic camel calves, indicating its role as one of the important bacterial agents associated with camel calf diarrhea. Preventive measures, including improved hygiene, adequate colostrum intake, and supportive fluid therapy, are essential for disease control (149). *Salmonella* spp. were identified as one of the major bacterial pathogens associated with diarrhea in camel calves, accounting for approximately 30% of the isolated bacterial species, besides *E. coli* (58%) and *Enterococcus* (12%) (9). *Salmonella* spp. represent important zoonotic pathogens in camels, contributing significantly to enteric disease and systemic infections, while their presence in both healthy and diseased animals underscore the need for improved hygiene, surveillance, and preventive management practices.

### 3.7 *Mycoplasma* spp

Mycoplasmas, belongs to class Mollicutes, in which *Mycoplasma* and *Acholeplasma* genera are included (150). *Mycoplasma* spp. is considered among the smallest self-replicating bacteria and are characterized by the absence of a rigid cell wall. They are instead bound by a trilaminar membrane like the conventional plasma membrane, placing them within the class Mollicutes. Despite their remarkably small genomes, which are among the most reduced in free-living organisms, mycoplasmas have evolved specialized strategies that facilitate intricate interactions with their hosts (151). Several studies have reported the occurrence of Mycoplasma infections in camel populations in different regions of the world. Since 1995, Mycoplasma has been recognized as one of the etiological agents responsible for respiratory disease outbreaks in camels in Ethiopia (152). In Saudi Arabia, the presence of *Mycoplasma* spp. in dromedary camels has been confirmed through molecular detection methods such as PCR (153). Similarly, in Egypt, studies investigating mycoplasmas in camels remain limited; however, the presence of *Candidatus Mycoplasma haemolamae* infection in camels has also been reported, indicating the occurrence of hemotropic mycoplasmas in camel populations (154). In Taif, Saudi Arabia, Mycoplasma infection was detected in 24 of 93 camel samples (approximately 26%) (153). *Mycoplasma arginini* has been isolated from pneumonic camels; 16 isolates were recovered from 100 camels with pneumonic lesions, corresponding to an isolation rate of 8.8%, and were associated mainly with chronic interstitial pneumonia that approved the link between the agent and the disease (155). Various species of *Mycoplasma* and *Acholeplasma* have been reported in camels, including *Mycoplasma arginini*, *Acholeplasma laidlawii*, and *Acholeplasma oculi*. In addition, serological evidence has indicated exposure of camels to other species such as *Mycoplasma mycoides* subsp. *mycoides*, *Mycoplasma capricolum* subsp. *capripneumoniae*, and *Mycoplasma mycoides* subsp. *Capri* (156). Mycoplasma DNA was detected in 10.3% of the samples examined. In camels, *Mycoplasma ovis-like* was identified in 2.9% of blood samples, representing the first reported detection of this organism in camel blood (157). Mycoplasmas are important but often overlooked pathogens in camels, with their diverse species and involvement in respiratory disease, alongside advances in molecular detection, underscoring the need for further research into their epidemiology, pathogenicity, and overall impact on camel health.

### 3.8 Pseudomonas aeruginosa

*Pseudomonas aeruginosa* is an opportunistic Gram-negative motile bacterium (158), and both pets and wild animals play a crucial role in the existence and circulation of high-priority *P. aeruginosa* (159). *P. aeruginosa* is linked to a wide range of diseases in animals, including infections affecting virous species, particularly respiratory tract infections. Microbial isolation from the respiratory tract of 98 apparently healthy camels in Egypt was conducted. Blood, nasal and tracheal swabs, and lung samples, collected from abattoirs in Cairo, Giza, and Elsharkia, Egypt revealing 77 isolates, including *Staphylococcus aureus* (14.5%), *Pseudomonas aeruginosa* ý (11.0%), *Bacillus* spp. (10.5%), *E. coli* (3%), and *Klebsiella* spp. (0.5%) (160). The isolation of *S. aureus* and *P. aeruginosa* from the respiratory tract of apparently healthy camels reflects their potential role in respiratory diseases encountered in camel populations (160).

Although *P. aeruginosa* is mostly opportunistic bacterium and recovered from healthy tissues, the horrific finding of antibiotic resistant strains in animal tissue is a burden biological hazard. *P. aeruginosa* was isolated from 45 healthy camel meat samples, with a prevalence of 22.5%. ESBL-producing *P. aeruginosa* was found in 45% (21/45) of the isolates from camel flesh (161, 162). The pathogen was highly resistant to rifampicin and ceftriaxone, then cefepime and aztreonam. *β*-lactamase gene prevalence percentages were *bla_PER-1* (28.5%), *bla CTX-M* (38%), *bla SHV* (33.3%), and *bla TEM* (23.8%) (161). Rifampicin- and Multidrug-Resistant *P. aeruginosa* have been detected in apparently healthy Camels (163). *P. aeruginosa* and *Klebsiella* spp. which cause diarrhea in ruminants and humans, can form biofilms encoded by biofilm-associated genes (*pelA* and *rmpA*) (164). *P. aeruginosa* was isolated from uterine swabs of camels (31.5%), cows (27.8%), mares (22.2%), and drinking water (18.5%), indicating that camels had the highest prevalence among the tested animals (158). In the eastern region of Saudi Arabia, *P. aeruginosa* is one of the most frequent bacteria linked to endometritis in cows, camels, and mares. *P. aeruginosa* high-risk clones *ST111, ST274, ST308, ST357*, and *ST446* have been found in endometritis-affected animals and their drinking water. The detection of the *blaTEM, blaSHV, blaCTX-M, and blaVIM* genes in the plasmid DNA highlights how antimicrobial resistance genes spread horizontally (55). *P. aeruginosa* is a significant opportunistic pathogen in camels, characterized by notable prevalence, virulence, and antimicrobial resistance. Its involvement in respiratory and reproductive infections highlights the importance of strengthened surveillance, effective monitoring, and improved control strategies.

### 3.9 Klebsiella pneumoniae

*Klebsiella pneumoniae* is an opportunistic Gram-negative pathogen associated with various infections, such as urinary tract infections, bacteremia, pneumonia, and liver abscesses (165). The pathogen is ubiquitously found on the mucosal surfaces of animals or in the environment such as water, soil, and other ecological niches. *K. pneumoniae* has recently become a pathogen of increasing clinical concern, due to the growing number of severe and difficult-to-treat infections. Based on the evolutionary diversity of clinical strains of *K. pneumoniae*, it is believed that many infection models, including pneumonia, liver abscess, and gastrointestinal colonization may represent well-recognized disease manifestations associated with this pathogen (165).

*Klebsiella pneumoniae* isolates has been identified as a significant cause of pneumonia and sudden death in neonatal dromedary camels (166), with some cases exhibiting severe lesions and poor responses to antimicrobial therapy (167). Pneumonia was reported in four neonatal camels (5 to 10 day- old) from a single farm. Affected camels showed weakness, fever, off-suckling behavior, respiratory distress, and sudden death. Bronchopneumonia was noted in two cases, while bronchointerstitial and interstitial pneumonias were each noted in one case. Isolation of *K. pneumoniae* was done from the lungs of all four cases and was confirmed by PCR (166). Comparison of phenotypic characteristics and virulence traits of *K. pneumoniae* isolated from pneumonic and healthy camels revealed that strains from pneumonic lungs were able to produce indole, urease positive, and exhibited hypermucoviscosity trait (168).

In Egypt, the predominance of *Klebsiella* species among camels was documented, and some virulence genes were screened. Camels exhibited clinical signs including respiratory manifestations, inappetence, or pyrexia, as well as profuse nasal secretions, and depression. *Klebsiella species* were prevalently isolated 36.8% (127 out of 345 samples) from nasal swabs (159), lungs (90), trachea (40), intestine (30), and liver (25) (169). By PCR, *iutA, fimH*, and *traT* genes were detected in all isolates (100%), whereas the *mrkA* gene was detected in 85% of strains. *Klebsiella* species have been identified as significant circulating pathogens within camel populations, possessing multiple virulent genes that could be linked to the rising incidence of respiratory issues in camels (169). In Iraq, *K. pneumoniae* exhibited a higher infection rate and was the predominant bacterium isolated from pneumonic camels at Al-Muthanna abattoir (170). The immune systems of camels that are less exposed to antibiotics are crucial in preventing *K. pneumoniae* from developing antibiotic resistance (171). In KSA, isolation of *K. pneumoniae* from diseased and apparently healthy farm animals (cows, sheep, goats, and camels) was done for recognition of *K. pneumoniae* subspecies from the Taif area. The incidences of *K. pneumoniae* subspecies in examined animals were markedly varied, being greater among diseased animals (25.2%) as compared with apparently healthy ones (5.5%) (172). *K. pneumoniae* is an emerging and clinically important pathogen in camels, particularly in neonatal infections, where its virulence traits and growing resistance to antimicrobial treatments emphasize the need for enhanced surveillance, prompt diagnosis, and responsible antimicrobial stewardship. Table 4 summarizes the reported pathogenic bacteria isolated from pneumo−/ enteritis in camels in Saudi Arabia.

**Table 4:** Summary of the reported pathogenic bacteria isolated from pneumo−/ enteritis in camels in Saudi Arabia.

Bacterial pathogen	Type of sample	Study population	Diagnostic method	No. of samples	No. of positive	Reference
*P. multocida*	Nasal swabs	pneumonic alive and dead camel calves	bacteriological isolation	48	48	(114)
*E. coli*	Faecal samples	diarrheic camel	bacteriological isolation	170	99	(35)
*E. coli*	Faecal samples	diarrheic camel calves	bacteriological isolation	190	52	(115)
*E. coli*	Cecal content	healthy camels	VITEK 2 real-time PCR	100	26	(126)
*E. coli*	Feces	Slaughterhouse camels	Bacterial culture and identification	206	5	(124)
*E. coli*	Hide	Slaughterhouse camels	Bacterial culture and identification	206	6	(124)
*K. pneumoniae*	Fecal Nasal swab Milk	Diseased and apparently healthy camels	Bacterial culture and identification	38 38 33	40% 15% 5.6%	(172)
*Pseudomonas aeruginosa*	Uterine swab Drinking water	Camels with clinical endometritis Camel herds	Bacterial culture and identification	60 30	17 3	(158)
*C. perfringens*	Undeclared Faecal swab Gastrointestinal samples	Camels with diarrhea Healthy and diarrheic dromedary camels She-camels with sudden death	ELISA Bacterial culture and PCR ELISA	121 463 340	26 262 51	(134) (137) (36).
*Mycoplasma* spp.	Nasal swabs	Camels	Semi-nested PCR and 16S rRNA gene sequencing	93	24	(153)

### 3.10 Polymicrobial infections contributed in pneumo−/ enteritis in camels

Although individual pathogen profiles provide important information on disease mechanisms, field outbreaks of camel pneumo-enteritis are increasingly recognized as polymicrobial syndromes involving interactions between viral and bacterial agents (10, 160). Available investigations of diseased camel calves have reported mixed pathogen detection in a substantial proportion of clinical cases, with co-isolation rates varying among studies according to geographic location, diagnostic methods, and herd management conditions. Bacterial agents cause of pneumo-enteritis in camels are *coliforms, staphylococci, Providencia* spp.*, Edwardisella* spp.*, Proteus* spp.*, Kluyvera, Pseudomonas* spp. *and Shigella*. A higher incidence of mixed infection (85.2%) and lower single infection cases (14.8%) is recorded and the most prevalent bacteria among respiratory affected cases are *S. aureus* (21.5% and from diarrheic camels, are *E. coli* (38.4%) (8). Viral infections such as PPRV, BVDV, and coronavirus infections may disrupt respiratory and intestinal mucosal barriers, impair innate immune responses, and increase bacterial adherence and proliferation, facilitating secondary infections by organisms including *Pasteurella multocida, Escherichia coli, Klebsiella* spp., and other opportunistic bacteria (4, 8). Also, Total of 1,200 calf-camels has morbidity rate of 240 (20%) in Taif, KSA and the morbidity is distributed in winter 130(54.2%) and in summer 110(45.8%). Mortality rates are 109(54.4%) from the scoured calf-camels, at winter 57(52.3%) and at summer 52(47.7%). The predominant isolated bacteria from scoured camel calves were *E. coli* (56.3%), *Clostridium perfringens* (53.3%), *Campylobacter jejuni* (29.6%), *Salmonella typhimurium* (18.8%), *Proteus vulgaris (*15.4%), *Enterococcus faecalis* (15%), *Brucella abortus* (14.2%), Enterotoxigenic *Escherichia coli* (9.6%) and *Yersinia entercolitica* (9.2%) (9, 173). Conversely, bacterial infections may intensify tissue damage, inflammatory responses, and systemic complications, contributing to increased morbidity and mortality compared with single-agent infections. Reports from Middle Eastern and neighboring arid camel-producing regions indicate that concurrent detection of multiple viral, bacterial, and parasitic agents is common in clinically affected herds, highlighting the need for diagnostic approaches that evaluate pathogen combinations rather than isolated agents (7, 8, 97). However, quantitative estimates of mixed infection frequency remain limited due to inconsistent surveillance protocols, emphasizing the importance of future longitudinal studies using multiplex molecular diagnostics to define pathogen interactions and their contribution to camel pneumo-enteritis severity. Table 5 summarizes the commonly reported combinations and clinical significance of polymicrobial infections associated with pneumo−/enteritis in camels.

**Table 5:** The commonly reported combinations and clinical significance of polymicrobial infections associated with pneumo−/enteritis in camels.

Infection pattern	Commonly reported combinations	Clinical significance	References
Viral–bacterial co-infection	Respiratory viruses + *Pasteurella multocida*, *E. coli*, *Klebsiella* spp.	Increased pneumonia severity, systemic inflammation, and mortality risk	(10, 35, 160)
Viral–viral co-circulation	PPRV, BVDV, coronavirus-related infections	Enhanced immune suppression and diagnostic complexity	(4, 23, 60, 96)
Bacterial–bacterial mixed infection	*Pasteurella* spp. with enteric/opportunistic bacteria	Exacerbated respiratory and intestinal lesions	(8, 11, 131, 152, 173)

### 3.11 Zoonotic significance of bacterial pathogens in camel pneumo−/enteritis

The most important zoonotic diseases are reported among Iranian camels including listeriosis, leptospirosis, plague, Q fever, brucellosis, campylobacteriosis, tuberculosis, pasteurellosis, clostridiosis, salmonellosis, *Escherichia coli* infections, rabies, camelpox, Middle East respiratory syndrome coronavirus, Crimean-Congo hemorrhagic fever, echinococcosis, cryptosporidiosis, toxoplasmosis and dermatophytosis, most of which belong to bacterial, viral, parasitic and fungal pathogens (174). Several bacterial pathogens involved in camel respiratory and enteric infections have important public health implications including *E. coli*, *Mycobacterium bovis, Pasteurella multocida* salmonellosis *and* campylobacteriosis. STEC including zoonotic strains associated with foodborne disease, represents a potential hazard through contamination of milk, meat, and the farm environment (123, 175). Similarly, *Mycobacterium bovis* infection in camels poses a significant One Health concern because of its zoonotic potential and implications for tuberculosis transmission through close animal contact and consumption of contaminated animal products (142, 145). Other respiratory bacterial agents, including *Pasteurella multocida*, may contribute primarily to animal disease but can occasionally cause opportunistic infections in humans (176). Therefore, pathogen surveillance in camel herds should incorporate antimicrobial resistance monitoring, food safety assessment, and occupational exposure evaluation to minimize risks associated with zoonotic bacterial infections. Table 6 summarized the regional prevalence, host age predilection, clinical presentation, main pathological lesions, and zoonotic risk levels of bacterial pathogens associated with pneumo−/enteritis in camels.

**Table 6:** The regional prevalence, host age predilection, clinical presentation, main pathological lesions, and zoonotic risk levels of bacterial pathogens associated with pneumo−/enteritis in camels.

Bacterial pathogen	Regional prevalence/occurrence	Host age predilection	Primary clinical syndrome	Main clinical lesions	Zoonotic risk level
*Pasteurella multocida*	Frequently reported in camel respiratory outbreaks in Middle Eastern and South Asian desert production systems, including acute pneumonic outbreaks	Mainly young calves and stressed animals after weaning or transport	Acute pneumonia, fever, respiratory distress, sudden mortality	Fibrinous bronchopneumonia, lung consolidation, pleuritis, septicemic lesions	Moderate (rare opportunistic human infections)
*Escherichia coli* (including STEC strains)	Commonly associated with camel enteric infections; STEC detection reported in food-producing camel systems	Neonates and young calves with inadequate passive immunity	Diarrhea, enteritis, septicemia; secondary respiratory complications possible	Enteritis, intestinal mucosal damage, dehydration, systemic inflammatory lesions	High for STEC strains (foodborne and environmental zoonotic risk)
*Klebsiella* spp.	Reported as opportunistic respiratory pathogens in stressed camel herds	Young, immunocompromised, or concurrently infected animals	Secondary pneumonia and mixed bacterial respiratory infection	Suppurative bronchopneumonia, airway inflammation, abscessation	Low to moderate (opportunistic zoonotic potential)
*Clostridium* spp.	Present in camel environments; disease associated with management stress and intestinal disturbances	Young animals and animals exposed to nutritional or environmental stress	Enterotoxemia, severe enteritis, sudden death	Necrotizing enteritis, intestinal hemorrhage, toxin-associated tissue damage	Low to moderate depending on species
*Mycobacterium bovis*	Reported sporadically in camel populations; importance linked to tuberculosis surveillance	Mainly adults, but infection possible at various ages	Chronic wasting disease, respiratory infection, reduced productivity	Granulomatous pneumonia, lymph node enlargement, granulomatous lesions	High (zoonotic tuberculosis risk)
*streptococcus pyogenes*	Sporadically in camel lungs, particularly in slaughterhouse surveys	No camel-specific age predilection	bacterial bronchopneumonia	suppurative or fibrinopurulent bronchopneumonia,	Low to moderate
*Salmonella* spp.	Reported mainly in Africa and the Middle East. Overall herd prevalence is generally low to moderate (approximately 5–10%), although higher isolation rates may occur during outbreaks or under stressful management conditions. Carrier animals are common.	Young calves (<6–12 months)	watery to hemorrhagic diarrhea, dehydration, weakness, and septicemia. Pneumo−/enteritis	fibrinonecrotic or hemorrhagic enteritis. Multifocal bronchopneumonia or fibrinous pneumonia secondary to septicemia	High. Infected camels may contaminate meat, raw milk, feces, and the environment.
*Mycoplasma* spp.	Reported mainly in North Africa and the Middle East. Respiratory mycoplasmosis is considered sporadic to endemic in many herds.	Camel calves (generally <1 year) showed severe illness, but adults appeared asymptomatic	nasal discharge (serous to mucopurulent), persistent cough, and reduced weight gain. In severe septicemic cases, pneumo−/enteritis-like syndrome. Polyarthritis, mastitis, keratoconjunctivitis, and abortions have also been reported with some *Mycoplasma* infections.	catarrhal to fibrinous bronchopneumonia, interstitial pneumonia, pleuritis, mild catarrhal enteritis	Low.
*Pseudomonas aeruginosa*	Middle East and North Africa in camels with respiratory manifestation	No camel-specific age predilection	bacterial pneumonia	usually opportunistic/secondary pathogen, but isolated from chronic proliferative bronchopneumonia and chronic pleuropneumonia.	Low for ordinary exposure, but potentially moderate/high in specific circumstances such as ARS and immunosuppression

## 4 Overview of parasites contributing pneumo−/enteritis in dromedary camels

### 4.1 *Eimeria* spp

*Eimeria* species are obligate intracellular coccidian parasites that inhabit the intestinal tract and are transmitted by ingestion of unsporulated oocysts shedded in the feces of infected animals (177). Among camelid *Eimeria* species, *Eimeria cameli* and *E. dromedarii* show the widest geographical distribution, whereas *E. bactriani*, *E. rajasthani*, and *E. pellerdyi* are confined to more limited geographical zones (178). Among these, *E. cameli* and *E. dromedarii* are the species most commonly associated with clinical coccidiosis (179). An epidemiological study on Mongolian Bactrian camels reported that the most prevalent *Eimeria* species were *E. cameli* (22.3%), *E. rajasthani* (37.3%), and *E. dromedarii* (27.7%). There is significant variation in infection patterns within camel populations, as evidenced by the fact that mixed infections were found in 24.8% of the analyzed samples whereas 39.0% of samples tested negative for detectable coccidian stages (180).

Coccidiosis occurs mainly in young animals, while adults are usually resistant because of immunity acquired after earlier exposure to *Eimeria* (181, 182). Young, infected animals and severe infections in adult camels exhibit diarrhea and hemorrhagic enteritis. Animals with serious infections exhibit inappetence, dehydration and progressive emaciation (183). The risk of mortalities in camel calves might be greatly increased by dehydration and subsequent bacterial infections (184). Camels that died from generalized weakness due to heavy *E. cameli* coccidiosis were highly emaciated, and most of them had passed bloody feces (178).

In a study conducted in Riyadh, *E. rajasthani* was identified in 30% of examined camel fecal samples using flotation test. Moreover, molecular analysis targeting the 18S rRNA and ITS-1 genes confirmed the usefulness of molecular tools for accurate species identification (185). In Al-Najaf, Iraq (2023–2024), *Eimeria* spp. were detected in 57% of examined camels using conventional PCR. Higher infection rates were observed in young male camels (<5 years of age), and histopathological findings revealed granulomatous and hemorrhagic enteritis with marked inflammatory cell infiltration and visible developmental stages of the parasite (186).

*Eimeria species* oocysts were examined in 200 fecal samples collected from slaughtered camel recti at the Cairo abattoir and were found in 38% of the samples examined. Three *Eimeria species* were identified: *E. cameli*-like oocysts in 31%, *E. rajasthani* in 18%, and *E. dromedarii* in 14% (187). Examination of 305 fecal samples from camels revealed 29 (9.51%) animals infected with *Eimeria* spp., with a higher prevalence recorded in winter (12.26%) compared to summer (6.67%), indicating seasonal variation in infection rates (178). Seventy-five (35.9%) of the 209 fecal samples taken from slaughtered camels at the West Abattoir in Riyadh and the Onaizah Modern Abattoir in Al-Qassim tested positive for *Eimeria* spp. (188). *Eimeria* species are common enteric parasites in camels, particularly in young animals, and their varying prevalence, clinical effects, and seasonal trends emphasize the need for effective management and accurate diagnostic approaches to reduce the impact of coccidiosis in camel herds.

### 4.2 *Cryptosporidium* spp

Cryptosporidium is a ubiquitous enteropathogen protozoan affecting livestock worldwide (189). Cryptosporidiosis is a significant zoonotic disease caused by Cryptosporidium spp., primarily transmitted via the fecal–oral route. The parasite induces pathological changes in many vertebrate hosts, including humans (190). *Cryptosporidium* is substantial gastrointestinal parasite that can contaminate drinking water streams with a lot of infectious oocysts (191). Infected hosts frequently develop persistent diarrhea as a primary clinical manifestation, making *Cryptosporidium* spp. one of the most important causative agents of chronic enteric disease (192). At present, cryptosporidiosis represents a major concern for animal and public health, as no effective pharmacological treatments or vaccines are available for its prevention and control (193). Cryptosporidium infection in camels is associated with severe watery diarrhea and dehydration, potentially leading to mortality in neonatal calves, in particularly severe cases. Furthermore, camels may act as reservoirs for zoonotic *Cryptosporidium* species (194).

Cryptosporidium infection in camels is pandemic and evidences for cosmopolitan nature of infection has been reported, including the United States (195, 196), Czech Republic (197, 198), China (199, 200), Iran (201, 202), Egypt (203, 204), Iraq (205), Saudi Arabia (206), Algeria (207, 208), and Australia (191). In a large-scale investigation, a total of 1,455 samples were examined, of which 126 tested positive for *Cryptosporidium* spp. Molecular analysis identified three species in camels, namely *C. andersoni, C. parvum,* and *C. occultus*, with *C. andersoni* being the predominant species (209). According to previous study findings, no significant associations were detected between *Cryptosporidium* infection and the age, sex, or season of the examined camels (210). In contrast, a recent molecular study in Egyptian dromedary camels reported that younger camels (<2 years of age) were significantly more likely to harbor *Cryptosporidium* infections, indicating an age-related risk factor for infection (204). *Cryptosporidium* was detected in 61 of 300 fecal samples (20.33%) and in 12 of 100 abomasal mucosa samples (12%), with simultaneous infections observed in three camels during summer (210). An overall prevalence of 10% for *Cryptosporidium* infection was recorded among camels examined in the Miandoab region (17/170) (189). Another study reported that, among 100 fecal samples collected from camels in Najaf Province, Iraq, the prevalence of cryptosporidiosis was 63.63% in males (33 samples) and 59.70% in females (67 samples) (205). *Cryptosporidium* spp. are globally distributed enteric parasites of camels with significant zoonotic implications, and their association with diarrheal disease, particularly in young animals, highlights the need for improved surveillance, water hygiene, and effective control strategies to limit their impact on animal and public health.

### 4.3 *Giardia* spp

Giardia spp. are one of the most common intestinal parasites worldwide, with millions of reported symptomatic infections, particularly in Asia, Africa, and Latin America (211). *Giardia duodenalis* has a two-stage life cycle consisting of a motile trophozoite that multiplies in the small intestine and an environmentally resistant cyst that is shed in feces and transmitted through the fecal–oral route (212). In livestock production, giardiasis has been linked to economic losses resulting from high treatment costs, mortality in young animals, and decreased weight gain (213).

Giardia infection generally leads to a self-limiting form of giardiasis, characterized by gastrointestinal disturbances including diarrhea, abdominal cramps, bloating, weight loss, and malabsorption. However, asymptomatic cases are frequently documented, particularly in developing countries (214, 215). *Giardia duodenalis* releases a protein called enolase when it attaches to intestinal epithelial cells. This protein helps the parasite attach to the cells and activates plasminogen, which contributes to tissue damage. As a result, enolase can cause injury to the intestinal lining, including cell separation and cell death through a necroptosis-like process. These results imply that enolase may function as a virulence factor and is crucial to the pathophysiology of giardiasis (216). *Giardia duodenalis* infection causes damage to the intestinal villi, inflammation, and edema, with parasites present in the intestinal lumen. The severity of tissue injury is greater in immunosuppressed hosts, indicating that immune status plays a key role in the pathogenesis of giardiasis (217).

*Giardia* commonly infects the intestinal tract of domestic animals, including livestock, dogs, and cats (218, 219), and wildlife (220). Among the species of this genus, *Giardia duodenalis* (also known as *G. lamblia* and *G. intestinalis*) is responsible for giardiasis in humans and a wide range of mammalian hosts, thereby classifying the disease as zoonotic (221). Molecular analysis revealed that *Giardia duodenalis* was detected in 16% of the examined camels, and the parasite was also identified in environmental samples collected from the same desert regions (208). A study reported clinical cases of giardiasis in dromedary camels (*Camelus dromedarius*) in Saudi Arabia, characterized mainly by severe intermittent watery diarrhea, poor appetite, and poor body condition. The infection was confirmed by the presence of *Giardia* trophozoites in fecal samples, and affected camels showed hematological alterations including low hematocrit and elevated leukocyte counts (222). Another study reported that the prevalence of *Giardia* infection, based on microscopical examination, was 20% (40/200 fecal samples (223). In Najaf Province, giardiasis was detected in 18.18% of male camels and 26.86% of female camels out of 100 examined fecal samples (205). Early documentation of giardiosis in dromedary camels (*Camelus dromedarius*) in Saudi Arabia provided some of the first clinical evidence of Giardia infection in this host species (222). *Giardia* spp. are widely distributed zoonotic intestinal parasites in camels, associated with gastrointestinal illness and economic losses. Their occurrence in both animals and the surrounding environment underscores the importance of improved hygiene, prompt diagnosis, and effective control strategies to limit their spread and impact.

### 4.4 Balantidium coli

*Balantidium coli* (*B. coli*) is a ciliate protozoan that commonly inhabits the intestine of the vertebrate species, such as ruminants, swine, non-human primates, and humans (224) (225). Although *B. coli* is usually a commensal organism in healthy humans and animals, it can become an opportunistic pathogen when the intestinal epithelium is compromised by other infections (226). The life cycle of *Balantioides coli* (formerly *Balantidium coli*) includes two stages: cyst and trophozoite. The trophozoite is the active and replicative form residing in the large intestine (227). In livestock, including camels, balantidiasis may contribute to economic losses by affecting growth, productivity, and reproductive efficiency (228, 229). A limited number of reports have documented balantidiasis caused by *B. coli* in less commonly affected mammalian hosts such as dogs, sheep, and horses (230). In addition, only a limited number of studies have reported the presence of *B. coli* in fecal samples from camels (231). However, recent molecular evidence indicates that the camel ciliate is classified as a distinct *Buxtonella* species rather than *Balantioides coli*, indicating that camels are unlikely to serve as reservoirs for human balantidiasis (232). Despite earlier suggestions that camels may act as potential reservoir hosts for *B. coli* in some regions (231), this hypothesis has been challenged by recent molecular reclassification. A study described a clinical case of balantidiasis in a dromedary camel presenting mainly with gastrointestinal signs, including soft, mucus-coated feces. Diagnosis was confirmed by rectal fecal examination using a saturated sucrose flotation method, which revealed a plenty of trophozoites and cysts *of B. coli* (233).

Balantidiasis may present as an asymptomatic infection or progress to severe dysentery characterized by watery, fetid diarrhea, anorexia, poor body condition, dehydration, impaired growth, and decreased production (234). A previous study found no significant association between infection and gender in camels, although gender was significant among breeders. Age was identified as a risk factor in both groups, while prevalence did not differ significantly between camels and male breeders, indicating a potential epidemiological relationship (235). In contrast, a study conducted in the Qassim region of Saudi Arabia reported significant differences in infection rates according to both age and sex among camels. The authors suggested that these findings may reflect regional epidemiological patterns and differences in management or environmental factors (236). A study reported the exclusive detection of *Balantidium coli* in camels suffering from diarrhea (237). According to earlier report, Balantidium coli has been identified in fecal samples from camels (238). A Saudi case report documented *Balantioides coli* infection in a young Arabian camel exhibiting anorexia, fever, and mucoid diarrhea, confirmed by fecal detection of trophozoites, underscoring its potential zoonotic importance (239). While *Balantidium coli* infection in camels appears to be relatively uncommon and often opportunistic, emerging molecular evidence and sporadic clinical cases highlight its potential role in gastrointestinal disease and the need for further investigation into its epidemiology and zoonotic significance.

### 4.5 *Dictyocaulus* spp. (lungworm)

Lungworms are parasitic nematodes classified within the *superfamilies Trichostrongyloidea* and *Metastrongyloidea*. The genus Dictyocaulus belongs to the *Trichostrongyloidea* and includes species such as *Dictyocaulus viviparus* and *Dictyocaulus filaria* (240). The genus *Dictyocaulus* currently comprises eight recognized species, each associated with specific animal hosts. These include *D*. *viviparus* in large ruminants such as cattle and buffaloes, *D. filaria* in small ruminants including sheep and goats*, D. arnfieldi* in equids, *D. eckerti* in cervids, *D. cameli* in camels, *D. africanus* in African artiodactyls, *D. capreolus* in roe deer, and *D. cervi* in red deer. Among these species, *D. viviparus* and *D. filaria* are the most prevalent worldwide and are responsible for significant economic losses and adverse effects on animal welfare (241). In the direct life cycle of D*ictyocaulus* species, infectious third-stage larvae (L3) develop in the environment after first-stage larvae (L1) are released in feces. After ingestion during grazing, L3 migrates to the lungs where they mature in the bronchi and bronchioles. Diagnosis is typically by using the Baermann technique for detection of L1 in feces (240). Host immune response against *Dictyocaulus species* infection induces bronchitis and eosinophilic pneumonia, and when combined with obstruction of the bronchiolar lumen, may result in coughing and dyspnea, fever, anorexia, poor growth, and increased mortality (242–244). Harsh lung sounds are evident on chest auscultation, while secondary bacterial infections may contribute to disease severity (245). While *Dictyocaulus filaria* and *Dictyocaulus viviparus* are typically associated with ruminant hosts, their occurrence has additionally been reported in camels (118). In a survey of internal parasites in *Camelus dromedarius*, *Dictyocaulus* spp. were detected among the nematode infections in camels, indicating the occurrence of lungworms in these animals (246). A recent review reported that *Dictyocaulus cameli* is a specific lungworm species infecting dromedary camel. However, there are still limited studies on its epidemiology and molecular characteristics, indicating the need for further research in this area (247). Lungworms of the genus *Dictyocaulus*, particularly *D. cameli*, are important parasitic agents in camels that can cause respiratory disease and economic losses. However, the scarcity of epidemiological data highlights the need for further studies to better understand their distribution, pathogenic effects, and effective control measures.

*Dictyocaulus cameli* represents an important parasitic component of camel respiratory disease complexes, particularly in arid and semi-arid pastoral systems in KSA and UAE. Infection occurs through ingestion of infective larvae from contaminated grazing environments, with transmission influenced by seasonal rainfall, grazing patterns, herd movement, and pasture contamination (248–250). *Dictyocaulus* spp. infection causes general illness, cough, polypnea, dyspnea, anorexia, and decreased productivity (251). Surveys conducted in camel populations of Pakistan, including Balochistan regions, have reported the presence of *Dictyocaulus* infections with variable prevalence rates depending on geographic location, climatic conditions, age group, and diagnostic approach. Higher infection rates have generally been associated with extensive grazing systems, communal watering points, and seasonal periods favorable for larval survival (252, 253). Clinically, lungworm infection may cause coughing, nasal discharge, respiratory distress, and reduced productivity; importantly, parasitic damage to bronchial tissues may compromise respiratory defenses and increase susceptibility to secondary bacterial pneumonia (252, 253). Therefore, *D. cameli* should be considered a potential contributor to the respiratory component of camel pneumo-enteritis, particularly when concurrent viral or bacterial infections are present.

### 4.6 Gastrointestinal nematodes (roundworms)

Nematodes are a broad group of roundworms that have a variety of eating behaviors, including parasitism of plants and animals. These parasites offer serious health risks to both humans and animals and result in large financial losses in agriculture (254). Most animal and plant species can be affected by one or more nematode species. In agriculture, nematode infestations lead to major economic losses worldwide, estimated at about $157 billion annually (255). A large epidemiological survey in central Saudi Arabia demonstrated the presence of several gastrointestinal nematodes in camels, including strongyle-type, *Nematodirus*, and *Strongyloides*. Infection was significantly associated with anemia and significant hematobiochemical alterations (256) Similarly, a field study in Ethiopia demonstrated a high prevalence of gastrointestinal nematodes in camels, with Trichostrongylus spp. being the dominant parasite. Ivermectin and albendazole showed strong efficacy in reducing nematode egg counts (257).

In another cross-sectional study conducted at Akaki Abattoir, Addis Ababa, Ethiopia, 55.5% of 384 examined camels were found positive for gastrointestinal nematodes. Strongyle-type eggs were the predominant parasites detected, and infection showed a significant association with poor body condition (258). A study conducted in central, western, and southern regions of Saudi Arabia reported the presence of several gastrointestinal nematodes in *Camelus dromedarius*, including *Nematodirus*, *Strongylus*, *Marshallagia*, and *Trichuris* (259). A comprehensive checklist of parasites infecting *Camelus dromedarius* in Saudi Arabia identified 13 nematode species, highlighting the wide diversity and epidemiological importance of gastrointestinal nematodes in camels under arid and semi-arid conditions (260). Gastrointestinal nematodes are widely prevalent among camel populations and play a significant role in compromising health and causing economic losses, emphasizing the need for routine parasite control, improved management practices, and expanded epidemiological research to reduce their overall impact.

Gastrointestinal nematodes are widespread parasitic infections affecting camel productivity and health, with epidemiological importance in pastoral herds of KSA and neighboring arid countries (261, 262). Regional surveys from Balochistan and other camel-producing areas in Pakistan have documented infections caused by several nematode genera, including *Haemonchus, Trichostrongylus, Nematodirus, Strongyloides, and Oesophagostomum species*, with prevalence influenced by season, husbandry practices, grazing intensity, and host age. Young camels are particularly vulnerable because of immature immunity, nutritional stress, and exposure during early grazing periods. Heavy gastrointestinal nematode burdens may result in diarrhea, weight loss, anemia, reduced growth, and impaired immune competence, thereby increasing vulnerability to concurrent respiratory and enteric pathogens (252, 253). In camel pneumo-enteritis cases, gastrointestinal nematodes may act as important predisposing factors by weakening host resistance and facilitating more severe outcomes following viral–bacterial infection. Despite their relevance, gastrointestinal helminths remain underrepresented in integrated camel disease investigations, and further studies are required to determine their contribution to mixed parasitic–microbial infections in diseased camel calves. Table 7 summarizes the reported protozoa and worms from cases of pneumo−/ enteritis in camels in Saudi Arabia.

**Table 7:** Summary of the reported protozoa and worms from cases of pneumo−/ enteritis in camels in Saudi Arabia.

Protozoan pathogens	Type of sample	Study population	Diagnostic method	No. of samples	No. of positive	Reference
*Eimeria spp*	fecal samples	Camels from the old camel market	Microscopy and PCR	150	45	(185)
*Eimeria spp*	Fecal samples	Slaughterhouse camels	Flotation microscopy	209	75	(188)
*Eimeria spp*	Fecal samples	Saudi Arabian camels	Microscopy (histopathological examination)	385	~160 (41.6%)	(181)
*Cryptosporidium spp*	Fecal samples	Diarrhoeic camel calves (calves < 3 months)	Not specified	33	10	(206)
*Cryptosporidium* spp.	Fecal samples	Diarrhoeic dromedary camel calves	Antigen-capture ELISA (Ag-ELISA)	92	16	(267)
*Balantidium coli*	Fecal samples	Dromedary camels of different ages	Faecal examination (microscopic detection of B. coli)	500	105	(236)
Roundworms	fecal samples	Dromedary camels	Microscopic examination	1,377	798	(259)
Gastrointestinal nematodes	Fecal samples	Dromedary camels	Faecal examination (parasitological examination)	1,195	~87 (7.28%)	(268)

### 4.7 Zoonotic significance of parasitic pathogens in camel pneumo-enteritis

Zoonotic parasitic infestation in camels has been reviewed by (98, 99, 174, 176). Camel echinococcosis is the most studied zoonotic parasitic infection affecting humans but *Toxoplasma gondii, Cryptosporidium* spp., *Fasciola* spp., *Trichinella* spp., and *Linguatula serrata* originating from camels are also considered as major public health risks (99). Parasitic infections associated with camel pneumo-enteritis are primarily recognized for their impact on animal health and productivity; however, several camel-associated parasites have potential public health relevance. Gastrointestinal parasites may contaminate grazing areas and farm environments, creating opportunities for environmental transmission and exposure among livestock handlers (16, 98). In addition, some protozoal infections with zoonotic potential, including *Cryptosporidium* species, may represent a concern through fecal contamination of water sources, food products, and shared environments (17). Balantidiasis and Giardiasis (141, 204, 215, 221, 239) are having zoonotic potentials from camels. Strengthening parasite surveillance in camel production systems using molecular identification methods is important for distinguishing animal-specific infections from zoonotic species and for improving both camel health management and public health protection.

## 5 Clinical presentation of camel pneumo-enteritis syndrome

Camel pneumo-enteritis is characterized by a complex clinical syndrome resulting from the combined effects of respiratory and enteric infections, with disease severity influenced by the type and number of co-infecting pathogens, host immunity, age, and environmental stress conditions (263). Although clinical manifestations may vary among outbreaks, affected camel calves commonly present with a combination of respiratory, gastrointestinal, and systemic signs rather than isolated organ-specific disease (7, 8).

The initial clinical presentation frequently includes fever, depression, reduced appetite, and decreased activity, reflecting systemic inflammatory responses associated with viral, bacterial, and parasitic infections. Respiratory involvement is typically manifested by nasal discharge, coughing, increased respiratory effort, dyspnea, and abnormal lung sounds. Nasal discharge may progress from serous to mucopurulent character as secondary bacterial infections develop, particularly following primary viral damage to respiratory mucosal defenses (6, 11, 76, 88, 113).

Concurrent intestinal involvement is a defining feature of pneumo-enteritis and is commonly observed as watery diarrhea, dehydration, abdominal discomfort, and reduced nutrient absorption. The combination of respiratory distress and enteric fluid loss can rapidly compromise the physiological status of affected calves. Persistent diarrhea, anorexia, and intestinal inflammation contribute to progressive weight loss, poor growth, and reduced body condition, particularly in recently weaned camels with limited immune reserves (35, 116, 121, 206).

In severe mixed infections, clinical deterioration may occur rapidly, with septicemia, systemic inflammatory responses, severe dehydration, and sudden death observed in highly affected animals (36, 173). Mortality risk is increased when viral infections, bacterial pneumonia, parasitic burdens, and environmental stressors occur simultaneously (9, 13). Therefore, recognition of the combined clinical pattern of fever, nasal discharge, watery diarrhea, weight loss, and acute mortality as well as ultrasonographic investigation of pneumo-enteritis revealing comet-tail artifacts and lung consolidation for pneumonia, and loop dilation alongside peritoneal fluid for enteritis (264, 265) are essential for early field diagnosis and implementation of appropriate control measures in camel herds. Table 8 summarizes the keystone gross, histopathological lesions and clinical significance of pneumo-enteritis in camels.

**Table 8:** Summary of the keystone gross, histopathological lesions, and clinical significance of pneumo-enteritis in camels.

Disease component	Gross lesions	Histopathological lesions	Clinical significance
Respiratory tract	Lung consolidation, congestion, edema, mucus/pus in airways, pleuropneumonia	Bronchointerstitial pneumonia, alveolar damage, inflammatory infiltration, epithelial necrosis	Respiratory distress, hypoxia, increased mortality
Intestinal tract	Congested/thickened intestine, catarrhal or hemorrhagic enteritis, fluid accumulation	Villous atrophy, enterocyte degeneration, crypt injury, inflammatory infiltration	Diarrhea, dehydration, weight loss
Lymphoid/systemic organs	Enlarged lymph nodes, splenic enlargement, organ congestion	Lymphoid depletion, systemic inflammatory changes	Immune suppression and disease progression
Mixed infection pattern	Combined pneumonia and enteritis with systemic involvement	Concurrent pulmonary and intestinal inflammatory lesions	Severe pneumo-enteritis syndrome and sudden death

## 6 Integrated gross and histopathological lesions associated with camel pneumo-enteritis

Pathological changes associated with camel pneumo-enteritis reflect the combined effects of respiratory and intestinal infection rather than the activity of individual pathogens alone. Because mixed viral, bacterial, and parasitic infections frequently occur under field conditions, lesion interpretation should consider concurrent damage affecting the respiratory tract, gastrointestinal system, and systemic organs (2, 7, 8).

### 6.1 Gross pathological findings

The respiratory tract commonly shows lesions consistent with severe pneumonia, including congestion and consolidation of affected lung lobes, cranioventral hepatization, pulmonary edema, fibrinous exudation, and accumulation of mucopurulent material within bronchi and airways. In severe bacterial complications, pleuropneumonia, abscess formation, and fibrin deposition may occur. Viral-associated respiratory injury may be characterized by diffuse pulmonary congestion, interstitial edema, and increased susceptibility to secondary bacterial invasion (27, 85, 95, 96).

Enteric lesions frequently include thickening and congestion of intestinal mucosa, catarrhal or hemorrhagic enteritis, excessive intestinal fluid accumulation, and enlarged mesenteric lymph nodes (1, 3, 173). Protozoal and bacterial infections may produce villous damage, mucosal necrosis, and impaired intestinal absorption, contributing to diarrhea and dehydration. In advanced mixed infections, systemic lesions such as enlarged lymph nodes, splenomegaly, hepatic congestion, and serosal inflammation may indicate generalized inflammatory responses or septicemic progression (116, 149, 206).

### 6.2 Histopathological findings

Microscopic examination of affected lungs commonly reveals bronchointerstitial pneumonia characterized by inflammatory cell infiltration, epithelial degeneration, bronchiolar necrosis, alveolar septal thickening, edema, and accumulation of inflammatory exudate within airways (10, 28, 97). Bacterial superinfection may result in suppurative bronchopneumonia, fibrinous inflammation, and extensive tissue destruction (3, 28).

Intestinal histopathology typically demonstrates villous atrophy, epithelial necrosis, inflammatory infiltration of the lamina propria, crypt damage, and variable degrees of enteritis depending on the causative agents (139). Parasitic infections may be associated with mucosal irritation, epithelial disruption, and inflammatory responses that further compromise intestinal barrier function (2, 266).

The combined presence of respiratory and intestinal lesions provides a pathological basis for the clinical syndrome of camel pneumo-enteritis, where disruption of mucosal barriers, immune suppression, and pathogen interactions contribute to increased disease severity and mortality. Table 9 summarizes of the pathogen’s keystone primary target organ, gross and histopathological lesions, and contribution to pneumo-enteritis in camels.

**Table 9:** Summary of the pathogen’s keystone primary target organ, gross and histopathological lesions, and contribution to pneumo-enteritis in camels.

Pathogen group	Primary target organ	Gross lesions	Histopathological lesions	Contribution to pneumo-enteritis severity
Viral agents	Respiratory/intestinal mucosa	Congestion, edema, mucosal injury	Viral cytopathic changes, necrosis, inflammation	Barrier disruption and predisposition to secondary infection
Bacterial agents	Lung, intestine, systemic organs	Consolidation, suppurative lesions, enteritis	Neutrophilic inflammation, tissue necrosis	Severe pneumonia, septicemia, mortality
Protozoal agents	Intestine	Enteritis, mucosal thickening	Villous injury, epithelial destruction	Diarrhea and immune compromise
Helminth parasites	Lung/intestine	Nodular lesions, airway, or intestinal irritation	Parasitic-associated inflammation	Chronic stress and increased susceptibility

## 7 Research gaps and future priorities in camel pneumo-enteritis investigations

Despite increasing recognition of camel pneumo-enteritis as a multifactorial respiratory–enteric syndrome, several critical knowledge gaps continue to limit effective diagnosis, prevention, and control in camel production systems. Current literature is largely composed of cross-sectional surveys and outbreak reports, with substantial variation in diagnostic approaches, sampling strategies, and pathogen detection methods (Tables 1, 4, 7). These limitations restrict accurate comparison of disease prevalence, pathogen interactions, and mortality risk across geographic regions.

### 7.1 Lack of standardized field diagnostic assays

A major limitation in camel pneumo-enteritis management is the absence of validated, standardized, and affordable field diagnostic platforms capable of simultaneously detecting viral, bacterial, and parasitic co-infections. Most available investigations rely on laboratory-based molecular assays, culture methods, or serological testing performed under research conditions, which are often inaccessible for pastoral camel farms. Development of rapid multiplex diagnostic tools integrating pathogen detection with host-response biomarkers is needed to enable early identification of mixed infections and guide targeted treatment decisions under field conditions.

### 7.2 Limited vaccination research and preventive strategies

Although vaccination programs have been extensively developed for several livestock species, camel-specific vaccine research remains limited. Few controlled trials have evaluated the efficacy, duration of immunity, optimal vaccination schedules, or protective immune responses against major respiratory and enteric pathogens affecting camel calves. Future studies should prioritize multivalent vaccine approaches targeting commonly co-circulating pathogens and should evaluate vaccine performance under real-world desert production conditions, where nutritional stress, climatic fluctuations, and mixed-species exposure influence disease susceptibility.

### 7.3 Insufficient multi-year longitudinal herd surveillance

Current understanding of camel pneumo-enteritis epidemiology is constrained by the scarcity of long-term longitudinal studies following camel herds across multiple seasons and production cycles. Most available data represent single outbreak investigations or short-term prevalence surveys, providing limited insight into pathogen circulation patterns, seasonal risk factors, age-related susceptibility, and the development of herd immunity. Coordinated multi-year surveillance programs incorporating clinical monitoring, environmental assessment, and repeated molecular characterization are essential to define transmission dynamics and identify early predictors of severe disease outbreaks.

Addressing these research gaps will require integrated One Health approaches combining veterinary epidemiology, molecular diagnostics, immunology, and herd management studies. Such efforts are essential for developing evidence-based prevention strategies and reducing the economic and health impacts of pneumo-enteritis in camel-producing regions.

## 8 Conclusion

Camel pneumo-enteritis represents a complex multifactorial syndrome in juvenile dromedary camels, resulting from interactions among viral, bacterial, and parasitic pathogens within the context of environmental stress, management practices, and host immune status. Although individual pathogens contribute to respiratory or enteric disease, the greatest clinical impact often results from combined infections that disrupt mucosal defenses, promote secondary bacterial invasion, and increase morbidity and mortality in camel herds. Improved understanding of pathogen interactions, lesion patterns, and epidemiological risk factors is essential for developing effective prevention and control programs.

From a field management perspective, reducing the burden of camel pneumo-enteritis requires practical interventions adapted to pastoral production systems. Effective colostrum management should be prioritized, including ensuring that newborn calves receive adequate quantities of high-quality colostrum during the first hours of life to improve passive immunity and strengthen early mucosal protection. Regular monitoring of calf growth, body condition, and early clinical signs should be integrated into routine herd management to identify susceptible animals before severe disease develops.

Veterinary and farmer-based prevention programs should also incorporate strategic seasonal parasite control, particularly before and during periods of increased pasture contamination and climatic conditions favorable for parasite transmission. Targeted deworming strategies, supported by fecal examination where available, can reduce gastrointestinal parasite burdens that compromise immunity and increase susceptibility to secondary respiratory–enteric infections. In addition, management guidelines for mixed-species herding should encourage separation of young camel calves from other livestock during high-risk periods, minimize overcrowding at watering and feeding sites, and improve quarantine practices for newly introduced or clinically affected animals to reduce cross-species pathogen transmission.

Future control strategies should combine improved field diagnostics, camel-specific vaccination research, longitudinal herd surveillance, and farmer education programs. A coordinated One Health approach involving camel owners, veterinarians, researchers, and public health authorities will be essential for reducing disease losses, improving welfare, and supporting sustainable camel production in arid regions.
